# How have researchers defined institutions, politics, organizations, and governance in research related to epidemic and pandemic response? A scoping review to map current concepts

**DOI:** 10.1093/heapol/czac091

**Published:** 2022-10-29

**Authors:** Austin Wu, Shivangi Khanna, Shelly Keidar, Laura Jane Brubacher

**Affiliations:** School of Population and Public Health, University of British Columbia, 2206 East Mall, Vancouver, British Columbia, Canada, V6T 1Z3; School of Population and Public Health, University of British Columbia, 2206 East Mall, Vancouver, British Columbia, Canada, V6T 1Z3; School of Population and Public Health, University of British Columbia, 2206 East Mall, Vancouver, British Columbia, Canada, V6T 1Z3; School of Population and Public Health, University of British Columbia, 2206 East Mall, Vancouver, British Columbia, Canada, V6T 1Z3

**Keywords:** COVID-19, Institutions, Politics, Organizations, Governance, Public Health, Health Crisis Response, Epidemic and Pandemic Response, Preparedness

## Abstract

In recent years, the literature on public health interventions and health outcomes in the context of epidemic and pandemic response has grown immensely. However, relatively few of these studies have situated their findings within the institutional, political, organizational, and governmental (IPOG) context in which interventions and outcomes exist. This conceptual mapping scoping study synthesized the published literature on the impact of IPOG factors on epidemic and pandemic response and critically examined definitions and uses of the terms IPOG in this literature. This research involved a comprehensive search of four databases across the social, health, and biomedical sciences as well as multi-level eligibility screening conducted by two independent reviewers. Data on the temporal, geographic, and topical range of studies were extracted, then descriptive statistics were calculated to summarize these data. Hybrid inductive and deductive qualitative analysis of the full-text articles was conducted to critically analyze the definitions and uses of these terms in the literature. The searches retrieved 4,918 distinct articles; 65 met the inclusion criteria and were thus reviewed. These articles were published from 2004 to 2022, were mostly written about COVID-19 (61.5%), and most frequently engaged with the concept of governance (36.9%) in relation to epidemic and pandemic response. Emergent themes related to the variable use of the investigated terms, the significant increase in relevant literature published amidst the COVID-19 pandemic, as well as a lack of consistent definitions used across all four terms: institutions, politics, organizations, and governance. This study revealed opportunities for health systems researchers to further engage in interdisciplinary work with fields such as law and political science, to become more forthright in defining factors which shape responses to epidemics and pandemics, and to develop greater consistency in using these IPOG terms in order to lessen confusion among a rapidly growing body of literature.

**Reflexivity Statement:** The majority of the authors who contributed to this research are women, who provided significant guidance in its development. The authors' prior background in public policy and the health sciences provided the authors with both familiarity and awareness of technical terminology and concepts in the reviewed articles. Furthermore, LJB's prior background in knowledge synthesis studies informed this research. While many members of the team are part of minority ethnic and religious groups, immigrants to Canada, or recent descendants of immigrants to Canada, we also recognize that we are all guests on the land upon which our work at the University of British Columbia's Vancouver campus was conducted, which is the traditional, ancestral, and unceded territory of the Musqueam people.

**Acknowledgements:** We would like to acknowledge the UBC Working Group on Health Systems Response to COVID-19 (https://governhealth.ca/) in assisting the development of our research by reviewing drafts and providing valuable comments. In addition, we want to thank Ursula Ellis, librarian at the University of British Columbia Woodward Library, for her assistance with the development of the research questions as well has her role in supporting early discussions and searches. We would also like to acknowledge Yoel Kornreich and Mahrukh Zahid for their assistance in the development of this research.

## Introduction

In response to the ongoing COVID-19 pandemic, government responses to control and manage the disease have varied both in terms of the public health and social measures enacted, as well as in the intensity of the implementation of measures (Hale *et al.* 2022). This has led to a variety of health and social outcomes where some jurisdictions were more successful in containing the disease, while others were less so – despite often implementing the same interventions at what appeared to be similar stages of disease progression. It is clear that contextual elements, such as population demographics, social arrangements, preparedness, infrastructure capacities, and citizen cooperation, among others, are contributors to determining the efficacy of epidemic and pandemic response (Berman *et al.* 2021). 'Upstream' or 'contextual' factors may determine how the processes producing public health responses work, for example in terms of how scientific evidence is used to drive action as well as communications and persuading the population to comply with recommended interventions (Berman *et al*. 2021).

Even before the COVID-19 pandemic, it has been argued that, in North America in particular, the focus of public health towards biomedical factors and interventions over the social structures of illness and disease – such as the actions and compositions of governments – has antecedents going as far back as the advent of modern germ theory and bacteriology in the late 19th and early 20th centuries. Public health historians have described this time, and much of the

20th century as well, as an era when public health academics and practitioners alike shifted away from collectively-aimed social reform and towards 'scientific and technical remedies' for individuals (Brandt and Gardner 2000), increasingly in the realm of clinicians and biomedicine rather than other fields (Fairchild *et al.* 2010; Jones *et al.* 2021). A 2018 review calling for more attention "taking account of context" (Craig *et al.* 2018) is a valuable source on this topic which provides a number of additional, relevant references.

Our research team at the author's institute has focused on several of these upstream, contextual determinants which shape public health responses to epidemics and pandemics such as COVID-19, characterized as Institutes, Politics, Organizations, Governance (IPOG). Our analytical framework focuses governance (as decision-making processes) at the interface between politics and organization, influenced by wider contextual factors and institutions, which are defined broadly as social norms and rules affecting the behavior of actors (see Brubacher et al. 2022 for a graphical representation of this framework).

However, prior authors have noted that these factors have been poorly defined, measured, and understood, even if they are given mention in passing. For example, previous research on governance in health systems more broadly has noted that 'the literature on health systems governance is still unfettered at large' (NTR, AM and PC 2019) and that governance has been 'an elusive concept to define, assess, and operationalize', resulting in an overall 'conceptual chaos' (Barbazza and Tello 2014). Indeed, David Levi-Faur in *The Oxford Handbook of Governance* (Levi-Faur 2012), describing governance more broadly, notes that the 'notion of governance … was rarely used and nearly incomprehensible before the 1980s, and that 'the origins, meanings, significance, and implications of the concept of governance are often disputed'. Additionally, literature focused on institutions (Everts 2013; Rocco, Beland and Waddan 2020; Béland *et al.* 2021; Huang 2021), politics (Brahmbhatt and Jonas 2015; Greer and Singer 2017; Fowler, Kettler and Witt 2021), organizations (Bardosh *et al.* 2017), governance (Jönsson and Jönsson 2012; Honigsbaum 2017; Huang *et al.* 2020; Choi *et al.* 2021), or a combination thereof in relation to epidemic and pandemic response, frequently utilizes these terms, though without clearly defining or operationalizing their use.

The purpose of conducting this research emerged when we found a paucity of COVID-19 and pandemic-related literature in the social, health, and biomedical sciences which situate findings in IPOG contexts beyond public health interventions and health outcomes. To this end, the following question has been posed for the conceptual mapping scoping study: In the context of epidemic and pandemic response, how have researchers previously defined IPOG factors? By addressing this question, we aim to enumerate, summarize diverse findings, and critically analyze prior definitions of these four terms in the context of epidemic and pandemic response, in order to create more conceptual clarity for these terms and to better inform a common base of knowledge and reference to draw upon in discourse around IPOG. This review also supports our current and future work using these terms in gathering evidence on them and analyzing their influence on public health responses in a wide range of jurisdictions.

**Methods**

This conceptual mapping scoping study is part of a broader multidisciplinary case study investigating the drivers of health systems' response to COVID-19 and other health emergencies through a framework developed at the author's institute by its interdisciplinary Working Group on Health Systems Response to COVID-19 (Brubacher *et al.* 2022). Although our research team developed *a priori* definitions and operationalizations of the terms institutions, politics, organizations, and governance, we have sought to clarify our own use of these terms and contribute conceptual clarity to the broader literature.

As such, this research follows guidance from the Joanna Briggs Institute (Peters *et al.* 2015) as well as the Preferred Reporting Items for Systematic Reviews and Meta-Analyses extension for Scoping Reviews Checklist in *Appendix A* (Tricco *et al.* 2018). A scoping review was chosen as an appropriate method to investigate the temporal and geographic scope of articles on IPOG and epidemic and pandemic response. More significantly, conceptual mapping is a more specific type of scoping review methodology (Anderson *et al.* 2008) that is very fitting for exploring the ways in which prior research has defined and operationalized the concepts of institutions, politics, organizations, and governance in our study.

*Search strategy*

The following four databases were searched, with the intention of capturing a wide variety of perspectives from medicine, the social sciences, as well as grey literature: JSTOR, PAIS, Web of Science, and Ovid Medline. This decision to capture a variety of interdisciplinary perspectives reflects our IPOG conceptual framework. Further, our search strategy was designed to capture literature across all geographies, including high-income and low- and middle-income countries. The search strategy was developed in collaboration with a librarian at the author's institute, as well as the broader Working Group, all of whom advised on databases to search *[Table 1]*. While some forms of grey literature, such as research reports, were captured through database searches, no additional systematic search of the grey literature was conducted.

Searches were conducted in English with restrictions towards articles and research reports if allowed by the database. There were no restrictions related to study design. Furthermore, the search language was written to limit the focus of the study towards responses to infectious diseases, and exclude responses to non-infectious health crises, such as the opioid crisis, which is often characterized as an epidemic as well (US Department of Health and Human Services 2021).

***INSERT TABLE 1***

The initial scope of the search was without date restrictions, and was bounded only by the day upon which the search was conducted in 2021. Due to functional limitations on some of the databases, searches on studies published prior to 2011 were limited to PAIS and Ovid Medline. The impetus for the lack of date restrictions on the search was to include literature about other pandemics and epidemics beyond COVID-19, such as H1N1 and SARS. An updated search was conducted on 8 April 2022, using the same search strings on the same databases, but with an updated date range from 27 July 2021 to 8 April 2022.

Results from each database were exported as .ris files and imported into *Covidence* systematic review software. Additionally, upon recommendation from senior members of the Working Group on Health Systems Response to COVID-19 at the author's institute, the following topically-relevant journals were hand-searched:

- *Public Administration Review*
- *Journal of Health Politics, Policy and Law*
- *Journal of Comparative Policy Analysis: Research and Practice*
- *Nature*
- *BMJ Global Health*

With one article each from *Public Administration Review* and *Journal of Comparative Policy Analysis: Research and Practice* ultimately being included in the final results.

*Eligibility and Screening*

Two reviewers independently screened the retrieved records for eligibility using *Covidence.* For Level 1 (title/abstract) screening, eligibility was guided by the following questions: (1) Does the article discuss at least one of Institutions (I), Politics (P), Organizations (O), or Governance (G)[1] in the context of public health crisis management or preparedness? (2) Is the article in English? Those articles meeting both criteria proceeded to Level 2 (full-text) screening. Sample research topics included/excluded based on these criteria are noted in *Appendix C.*

In Level 1 screening, we identified that our search strategy was highly sensitive – and therefore retrieved many articles – but not necessarily specific enough. To manage the scope of the research, and ensure we were meeting our research objectives, we further refined our eligibility criteria after Level 1 to be more stringent in our exclusion of articles not pertinent to our objectives. As such, Level 2 screening was guided by the following criteria: Does the article define AND use[2] at least one of the IPOG terms in relation to public health crisis management or preparedness? Reviewers discussed all conflicts until consensus regarding inclusion or exclusion was reached.

*Data Extraction and Analysis*

[1] As defined in our broader case study protocol (Brubacher *et al.* 2022) Institutions are defined as the formal and informal rules and practices that shape human interactions in a society giving rise to meaning, norms, and appropriateness of behavior; Politics as the arena in societies where power is assigned and distributed and where important influences on decision-making regarding health and other topics of public interest occur; Organizations refer to governmental public bodies, especially the organizations charged with public-health functions, as well as others in the health system; and Governance as the decision-making processes occurring at the interface between politics and organizations, conditioned by institutional norms.

[2] As in, uses the exact term(s) (Institutions, Politics, Organizations and/or Governance) or employs similar terms or concepts as those defined in our broader case study protocol (Brubacher *et al.* 2022).

Two independent reviewers extracted the following data from each included article: Authors/Year/Title; study location; disciplinary lens(es); methodology (quantitative/ qualitative/mixed), IPOG factor(s) defined and used; definition/operationalization of IPOG term(s) in the study; and the reported impact of the IPOG factor(s) on public health crisis management and/or preparedness *[Appendix D]*. Conflicts between reviewers were again resolved by discussion and consensus as to the extracted content.

Basic descriptive statistics (e.g. counts and proportions) were calculated across articles. Extracted data were analyzed thematically, using a constant comparative method (Braun and Clarke 2006). *QSR NVivo®* software was used for organization of codes and retrieval of coded excerpts from articles. A hybrid of inductive and deductive coding was used to identify and categorize excerpts from articles according to their use of IPOG terms, as well as subcategories of themes within each of those four terms. Inductive coding was used to separate excerpts in relation to observations by the reviewers, while deductive coding was used in mapping some excerpts onto previously noted definitions, in particular conceptions of governance used in the *Oxford Handbook of Governance.*

## Results

### *Temporal and Geographic Scope of Articles*

In total, 65 publications met the eligibility criteria and were included in the review (Figure 1): 16 involved discussion of institutions, 17 involved politics, 6 involved organizations, and 34 involved governance. Eight of the included publications (12.3%) discussed multiple terms[3]. A list of included articles can also be seen in in Table 2:

***INSERT FIGURE 1***
***INSERT TABLE 2***

Included articles were published between 2004 and 2022. The majority of articles were published in 2020 or 2021 (60%), or focused on the COVID-19 pandemic (61.5%). No differences were observed in how IPOG terms were conceptualized in relation to pandemics versus epidemics. A visualization of the chronology of included articles can be seen in Figure 2 and the disease of focus in Figure 3, keeping in mind that some articles discussed multiple diseases.

***INSERT FIGURE 2***
***INSERT FIGURE 3***

In addition, there was a wide geographic scope pertaining to the jurisdictions studied in the included literature. A large proportion of articles studied jurisdictions in Asia, Europe, or

[3] This accounts for why the breakdown of articles per term adds to more than 65 included articles.

international groups such as the United Nations (32.3%, 24.6%, and 33.8%, respectively), while jurisdictions in Africa were studied the least (9.2%). The distribution of article count by geographic region is seen below in Table 3, keeping in mind that some articles discussed multiple geographic areas.

***INSERT TABLE 3***

### *How were Institutions, Politics, Organizations, and Governance defined and operationalized in the literature?*

Articles defined and operationalized the concepts of IPOG in varied ways. Individual results for each term follow below. For each term, examples of definitions and operational usage from the literature are provided, along with descriptions and reasoning behind the sub-categorizations they have been placed in within a wider term.

#### *Institutions*

***INSERT TABLE 4***

The first three listed definitions are one of two broad categories in which the term 'institutions' was used in the context of epidemic and pandemic responses in the retrieved literature – a sense of 'institutions' as norms, rules, ideas, and processes within a system. The fourth definition is an example of the second category; the use of the term 'institutions' as a synonym for organizations, as well as the structures and systems those organizations exist within.

In the first sense, norms, rules, ideas, structures, and/or processes were made present through mechanisms of 'institutionalization', either informally through guiding actors' behaviours (Rosella *et al.* 2013; Kim 2015; Nohrstedt and Baekkeskov 2018; De Angelis and de Oliveira), or formally through structures such as taxation or constitutions (Rosella *et al.* 2013; Windholz 2020; West-Oram 2021; De Angelis and de Oliveira). Similarly, the concept of institutions as organizations could take place formally or informally – in the sense of the former, discrete organizations such as the Public Health Agency of Canada (PHAC), the Global Fund, WHO, state-owned enterprises, or the World Bank (Doyle 2006; Adeel *et al.* 2020; Fei); in the sense of the latter, as 'structure[s of] authority, attention, information flows, and relationships', informal counterparts to 'formal executive, legislative, and bureaucratic structures of public health' (Rosella *et al.* 2013; Yan *et al.* 2021). The intermixing of the terms 'institutions' and 'organizations' to refer to the same concept especially highlights the fluidity in which these terms have been defined by various authors. For example, the prior practice of organizational mapping has taken place with only reference to 'institutional structure[s]', rather than 'organizations' (Schwartz, Evans and Greenberg 2007; Liu *et al.* 2021).

#### *Politics*

In the examined articles, a strict definition of politics, as well as a conception of what 'good politics' might be, was found in only one article (Lee 2020), even though 17 articles ultimately included in the extraction discussed politics. In this article, 'politics' on its own, without any prefixes or qualifiers, was defined as 'the relationships within a group or organization that allow particular people to have power over others' and 'how decisions are made within such groups or organizations including how shared goals are agreed and achieved'. From there, the notion of 'good politics in global health' is described as 'adherence to principles of good governance; that is, the extent to which rules and procedures are built on principles of transparency and accountability, effectiveness, representativeness and participation, and rule of law'.

The last characterization especially aligns with the more commonplace conceptions or characterizations of politics in relation to epidemic and pandemic response seen in the captured literature which focused on politics. Such articles fall under two broad categories: descriptions of what structures comprise the domain of politics and discussions of how they function in practice– legislative committees, political parties, drafting of legislation, partisan behavior – and value-based assessments of these structures and practices, such as 'good politics' and the relationship between power and agency over one's death.

Returning to this conception of what 'good politics' might be, as mentioned previously (Lee 2020), it is clearly stated in the same article that the 'spaces' in which politics take place are 'making (legislature), implementing (executive), and enforcing (judiciary)'. Other articles similarly describe places which the authors conceive of as spaces for politics to take place -- legislative action (Teigen 2007; Adeel *et al.* 2020; Afsahi *et al.* 2020; Nelson 2021), participation in a political system (Burkle 2020), the composition of a legislative committee (Moulds 2020), as well as partisanship and coalition-forming between political parties (Fowler, Kettler and Witt 2021; Nelson 2021).

In the context of epidemic and pandemic response, the concept of politics as values in part took the approach of describing the regulation of life and mortality as a political matter (Teigen 2007; Ragozina 2020; Biehl, Prates and Amon 2021). Of note here is the continued association of politics with power in this characterization, such as defining 'necropolitical' as the subjugation of 'life to the power of death' (Biehl, Prates and Amon 2021) and the way in which 'particular groups and communities are relegated to zones of living that are not life-giving but conditions of slow death … either through poverty, detrimental working conditions, nutrition, and pollution (Sandset 2021), as well as the Foucauldian notion of biopolitics, and by extension, biopower. Biopolitics in the captured literature is defined in the sense of states governing mortality in times of disease – through gaining authority to assign subjects into statuses of infected or uninfected (Lawson and Xu 2007), the 'moralization of health' through measures aimed at individuals, such as vaccine uptake (Hier), as well as 'the political regulation of corporality' and individuals' bodies through disease control measures (Ragozina 2020).

*Organizations*

Out of the four terms examined in this conceptual mapping scoping study, 'organizations' had the smallest number of articles captured in the search, a total of six. In most of these articles, analysis of the structure and functions of organizations involved within a public health response, such as non-governmental organizations (NGOs), government agencies, or components within agencies involved a form of organizational mapping of health systems (Liu 2004; Schwartz, Evans and Greenberg 2007), emergency response structures (Forestier, Cox and Horne 2016; Kim, Oh and Wang 2020; Liu *et al.* 2021), and the ways in which these structures have changed over time (Schwartz, Evans and Greenberg 2007; Kim, Oh and Wang 2020). This was akin to one of the definitions of governance proposed by Levi-Faur, that of governance as structure: for example, 'horizontal' or 'vertical' arrangements (Barbazza and Tello 2014), 'vertical integration' of health system components is also described as a type of 'organizational strategy' for reform (Liu 2004).

Relative to other terms, especially 'governance' and 'institutions', strict definitions for the term 'organizations' itself were sparse in the included literature. More frequent were descriptions of specific examples of organization*s:* an epidemic prevention station, county health bureaus (Schwartz, Evans and Greenberg 2007), aid groups such as the United Nations Development Programme (UNDP) (Forestier, Cox and Horne 2016), or even formal groups such as the Global Parliament of Mayors (GPM) with 'established patterns of communication, policy-making and exchange' (Boyce and Katz 2021). One included article was able to meet the previously listed conditions – organizational mapping of changes to a health system over time, describing the role and functions of each component and their relationship to each other, and placing the system in the context of its response to an infectious disease outbreak – while using the term 'organization' exclusively in reference to the WHO (Schwartz, Evans and Greenberg 2007).

*Governance*

***INSERT TABLE 5***

The use of the term 'governance' in the included literature was both the most numerous out of the four terms – with 34 articles (52.3%) providing a definition and operationalization of the term – and also the most varied in the ways in which researchers have previously defined these terms. We decided to categorize definitions of governance used by their scale of complexity, ranging from perceptions of governance as the structure of organization(s) involved in the response to a public health emergency, distinct consideration of processes within those structures as part of one's conception of governance, to perceiving governance as changes in structure and processes as part mechanisms to improve responses which could include broader reforms to structure and processes as an overall strategy guiding a jurisdiction's epidemic and pandemic response.

References to structures of governance alone in the captured literature are comparatively limited, and are largely focused on higher-level complex organizations such as the WHO (Carney and Bennett 2014), frameworks such as the IHR (Collins *et al.* 2021), or the structures of government hierarchies within national jurisdictions (Liu, Zhong and Yu 2020; Wang *et al.* 2021; Finnane). A description of a hypothetical governance structure to be used in a public health emergency can be seen in a proposed structure for governance during public health emergencies, in the form of an 'emergency republic' comprised of an independent technocratic agency of advisory experts akin to the American Federal Reserve, the relegation of the legislature to monitoring government accountability for the executive, and the passage of emergency powers from the executive to the independent technocracy (Gerwin 2011). The similarities between this conception of governance as structures alone means that many other articles which discuss similar content go by other names, such as 'organizations' and 'institutions', also discussed in this research.

In the included literature which conceptualized governance as processes within those structures, organizations such as the World Influenza Center (WIC) (Kamradt-Scott 2013) or the Association of Southeast Asian Nations (ASEAN) (Wenham 2018) as well as agreements such as the International Health Regulations (IHR) (Wilson and Lazar 2006; Gostin and Katz 2016; Zhou *et al.* 2020; Collins *et al.* 2021) were also discussed. However, in addition to descriptions of the existence of such structures or their base setups, articles in this category provided further context as to the interactions of various processes with one another, such as the ways in which subnational surveillance, national assessments, and WHO assessments interact with one another in the WHO director-general's decision to declare a Public Health Emergency of International Concern (PHEIC), visualized on a flow chart (Gostin and Katz 2016). An article on pandemic legislation in the European Union defined governance as 'the assignment of authority and the specification of procedures' (Speakman, Burris and Coker 2017).

Many articles also sought to describe processes of governance within the jurisdiction of a single state in the context of a single disease at both national and subnational levels of government. Processes characterized as 'governance' by their authors include matters such as collaboration and coordination between local, state/provincial, and federal/central levels of government in the United States and China respectively during the COVID-19 pandemic (Zhang and Zhang 2020; Liu *et al.* 2021), the 'preparedness or analytical capacity, coordination, regulation and implementation or delivery capacity' of Norway's government in response to COVID-19 (Christensen and Lægreid 2020), the use of enabling acts in transferring legislative power to an executive in times of emergency (Meszaros 2021), as well as the use of technology as a method by which to practice the act of governing and exercise authority, particularly through the use of online technologies to achieve societal functions remotely during responses to a disease (Nguyen 2009; Shultz and Melody 2021).

Governance in the included literature was also seen as including procedures of decision-making, such as ability to issue commands in a hierarchy. This is seen particularly in multilateral organizations such as the WHO (Carney and Bennett 2014; Agartan, Cook and Lin 2020) and the particular context of strong top-down centralized governance mechanisms in the People's Republic of China (He, Shi and Liu 2020; Zhang and Zhang 2020; Zhang and Wang 2021). The included literature describes changes in processes for decision-making in epidemic and pandemic response over time: for example moving away from exchanges and deliberation between jurisdictions towards 'member states ceding 'a considerable part of their respective sovereignty in national public health policy to the international community'' to increasingly structured and litigated groups such as the WHO (Wilson and Lazar 2006). One article characterized the WHO's role in global health governance as 'hegemonic' due to near-universal participation in it by the countries of the world, its displacement of states as the exclusive authority in certain international affairs, and the ways in which WHO-related instruments such as the IHR instruct member states to act in regards to infectious disease control, albeit without enforcement mechanisms (Hoffman 2010).

Values-based assessments of governance structures, processes, and mechanisms are used in some literature, such as descriptors like 'good governance'. In the context of epidemic and pandemic response, these frequently relate to exceptional conditions of governance during times of pandemic, going beyond 'normal' governing processes, and include an implied positive valuation with outcomes in the case of adoption of approaches for governing for the purposes of bolstering a jurisdiction's sense of security (Hanrieder and Kreuder-Sonnen 2014; Calain and Abu Sa'Da 2015; Wenham 2018).

The ways in which the local historical context of jurisdictions inform responses to health crises is mentioned as well and given an alternative values-based characterization as something other than 'good governance'. For example, both Vietnam and China are noted to employ a 'Leninist mode of governance' in their response to HIV/AIDS and COVID-19 respectively (Hirsch *et al.* 2015; He, Shi and Liu 2020); Chinese response to COVID-19 is especially noted in its use of militaristic rhetoric and fast-tracked promotions for well-performing local Communist Party (CPC) cadres involved in response. Both practices date back to the foundation of the People's Republic on the basis of armed revolution, and in particular, the latter practice of fast-tracked promotions for civilian cadres has a basis in 'battlefield promotions' for military personnel when the CPC was not yet in government in China prior to 1949 (He, Shi and Liu 2020).

A modality of governing particularly worth mentioning because of its direct relation to other works which provide discrete definitions for terms is the Foucauldian notion of 'governmentality' (Foucault 1991). In the context of epidemic and pandemic response, this was often used often used in the included articles in relation to the application of technology and the 'managerial and administrative capacities' of governments towards managing human life, as well as the discrete functions of states during a pandemic such as online learning technologies and the administration of social welfare (Makarychev and Romashko 2021; Shultz and Melody 2021). In particular, his concept of 'governing knowledges' discusses a philosophy of governing based primarily on practice, action, and technology, rather than theory or ideology (Shultz and Melody 2021). In all, five of the included articles used the framing of 'governmentality' in the context of governance during pandemics and epidemics (Lawson and Xu 2007; Nguyen 2009; Vankovska 2020: 19; Makarychev and Romashko 2021; Shultz and Melody 2021).

Also falling under the purview of governance strategies would be the practice of using values such as ‘democratic’, ‘smart’ or ‘good’ in defining ideal practices of governance, as noted in previous reviews on health governance more generally (Barbazza and Tello 2014). Articles in this review utilized concepts such as ‘adaptive governance’ (Kim *et al.* 2020), ‘global social governance’ (Agartan, Cook and Lin 2020), ‘authoritarian governance’ (Thomson and Ip 2020), ‘community public health crisis governance’ (Wang *et al.* 2021), ‘multi-level governance’ (Liu *et al.* 2021; Yao *et al.*) and the aforementioned ‘good governance’ (Coco and Dias 2020; Kim *et al.* 2020; Lee 2020). Definitions of these values ranged from the descriptive, such as ‘adaptive governance’ being defined as ‘flexible and learning-based multi-level modes of governance or institutional arrangements that can build resilience for the challenges posed by complex and urgent problems’, to the tautological, where the ‘principles of good governance’ were used as measurements against the notion of ‘good politics’ (Lee 2020).

Finally, it is worth noting here a tendency for reviewed articles to preface definitions with a prefix or qualifier, either to convey values (such as ‘good’ governance) or describe a particular typology, such as a horizontal or vertical structure of governance (Barbazza and Tello 2014). Although this took place most frequently with governance – where the value-based conception of ‘good governance’ has appeared frequently (Coco and Dias 2020; Kim *et al.* 2020; Lee 2020) along with descriptors such as ‘emergency’, ‘adaptive’, ‘administrative’ governance, and so on (Wilson and Lazar 2006; Hanrieder and Kreuder-Sonnen 2014; Agartan, Cook and Lin 2020; Kim *et al.* 2020; Thomson and Ip 2020; Liu *et al.* 2021; Wang *et al.* 2021; Zhang and Wang 2021) – this tendency has also appeared in regards to the terms ‘institutions’ and ‘politics’.

**Discussion**

*Rapid and recent growth in the literature and bias towards higher-income countries*

Although the search had no limitation on the lower end of the date range, we still found that a majority of the reviewed literature on IPOG in epidemic and pandemic response was related to COVID-19 (61.5%), or published in 2020 or 2021 (60%). In prior years, smaller upticks in publication of relevant literature were witnessed congruent with the global spread of H5N1 influenza in 2006/2007 and the 2013-2016 Ebola epidemic in Western Africa, but the cascade of literature related to COVID-19 exceeds both by a very wide margin. Indeed, by late summer of 2021, published literature on COVID-19 has exceeded that of H1N1, Zika, Ebola, HIV/AIDS, and even tuberculosis (Ioannidis *et al.* 2021).

Additionally, it was also noticed that the majority of the reviewed literature had a focus on multilateral groups such as the WHO (33.8%) or higher-income jurisdictions in Europe (24.6%) and Asia (32.3%), while articles which had a focus on jurisdictions in Africa in particular were comparatively scant (9.2%), especially given the disproportionate burden of disease still faced in many parts of the world (GBD 2019 Diseases and Injuries Collaborators 2020). To some extent this may be another side-effect of the enthusiastic academic response to COVID-19; a heavy focus on European countries in particular has been noted in prior writing on the impact of politics on jurisdictions' response to COVID-19 (Wu 2022). Furthermore, the origin of COVID-19 in East Asia, along with its early spread to Europe before other parts of the world (Sanyaolu *et al.* 2021), might explain the greater degree of attention given to these two regions of the world in the literature at the time of search.

*Institutions*

The dual definitions employed for the same term, 'institutions' – on one hand a synonym for organizations, and on the other as a term to capture a sense of norms, rules, ideas, and processes within a system – serve as a suitable initial indication of the fluidity in which these four terms -'institutions', 'politics', 'organizations', and 'governance' - were found to have been used in relation to each other in the context of epidemic and pandemic responses. In this particular case, what was characterized by some authors as a function of 'institutions' could just as easily describe functions of organizations or governance if using the terminology of a different author, or vice versa.

The use of the term 'institutionalization' may be seen as exemplifying this blurring of conceptual aspects of 'institutions', such as norms, with the 'structures' societies put in place to enable or support the realization of institutions. These 'structures' are often 'organizations' in actuality. For example, respect for the rule of law may be seen as an institution, while the courts are organizations which enable the rule of law to be applied, but they are not themselves the institutions per se. This distinction may be useful when research identifies conflicts or gaps between an 'institution' and the 'organizations' which exist to support it – for example, in a situation where courts are corrupt or perceived as not respecting the rule of law. Clarifying such distinctions in both defining these terms and framing them in relationship to each other could be useful in reducing confusion and providing clearer lines of discussion between publications.

*Discussion on politics*

Compared to other terms, and especially relative to the degree of discussion on the topic, precise definitions of 'politics' were difficult to come by. Indeed, some authors seemed to perceive 'politics' as comparatively fluid or subjective by contrasting it with practices perceived as more immutable or steadfast by comparison, for example law, science, or decisions made by agencies comprised of professional civil servants such as the Public Health Agency of Canada (PHAC) (Adeel *et al.* 2020; Dostal 2020; Liu, Zhong and Yu 2020), thus evading establishing a firm definition for 'politics' itself. In his apprehension for defining obscenity, United States Supreme Court Justice Potter Stewart famously wrote 'I know it when I see it' (*Jacobellis v. Ohio* 1964). In many ways the authors of the captured studies have attempted to use such intuitiveness to define 'politics' as well, perhaps attempting to bank upon the ubiquity of the term in daily conversation. However, in practice, the summation of such elusiveness was a sense of reluctance in the literature to define the term outright.

This elusiveness in defining 'politics' could also be seen in the way in which politics was characterized as a sense of values in the captured literature; for example, through characterizing the extent to which a conception of politics adhered to certain values, norms, or processes, and by extension act as ‘good politics’. Examples of some of these include ‘principles of transparency and accountability, effectiveness, representativeness and participation, and rule of law’ (Lee 2020), ‘legitimacy, transparency, accountability, equity, justice, and effectiveness’ (Forum on Medical and Public Health Preparedness for Catastrophic Events *et al.* 2016), as well as ‘social justice and equity’ (Liu, Zhong and Yu 2020). In some articles, while politics was not defined unto itself, ‘politics’ was contrasted against practices such as science (Liu, Zhong and Yu 2020), ‘the codified legal order’ (Dostal 2020), or ‘good governance’ (Lee 2020). There is a sense here that some authors have viewed politics as lacking an inherent value on its own, or even morally suspect, whereas others might see it as a way by which societies structure the processes around the use of power. Discussion of ‘politics’ was not the only situation in which descriptors of value were used; such tendencies were also seen frequently in regards to governance.

Another way in which writers sought to qualify their use of ‘politics’ in relation to epidemic and pandemic response was through the use of prefixes and qualifying terms, either attached to ‘politics’ – such as ‘good politics’ or ‘emergency politics’ (Afsahi *et al.* 2020; Lee 2020; Ragozina 2020) – or through the use of ‘politics’ as a qualifying term itself, like ‘political systems’ or ‘political participation’ (Burkle 2020). An especially common prefix term used in relation to ‘politics’ was the Foucauldian term ‘biopolitics’ (Lawson and Xu 2007; Ragozina 2020; Vankovska 2020; Biehl, Prates and Amon 2021; Makarychev and Romashko 2021), and by extension, biopower (Nguyen 2009; Ragozina 2020) and modes of objectification (Lawson and Xu 2007).

In defining biopower and biopolitics, Foucault writes of the former as ‘an explosion of numerous and diverse techniques for achieving the subjugation of [physical, biological] bodies and the control of populations’ (Liesen and Walsh 2015), and the latter as the mechanism by which biopower acts – ‘the processes by which human life, at the level of the population, emerged as a distinct political problem’, and the extension of states’ power over the bodies of a population, physical and political alike (Means 2021). In comparison to other articles with a focus on the politics of pandemics, the invocation of biopolitics and biopower is very much focused upon the values of politics rather than the structures it may take, such as legislative coalitions or the leadership of political parties.

We note from the uses of ‘politics’ in the literature we reviewed again some blurring between processes of power relationships and the organizations in which these processes occur or are expressed, such as legislatures and political parties. As an example, again using the distinctions previously set up in discussion on ‘institutions’, a legislature could be seen by itself as not ‘politics’, but instead ‘politics’ as an important determinant of what the legislature does or does not do. In our framing, these processes of where politics and organizations interact is where we locate ‘governance’.

*Governance*

Although writing on governance comprised the largest proportion of the reviewed literature (36.9%), in many ways it was also the most varied of the four analyzed terms. Contingent upon the terminology used by the authors, writing on governance could similarly be applied to organizations, institutions, and politics alike without mention of either term, further underscoring the fluidity in which these four terms have been used in relation to each other in practice.

An example of 'governance' being used in relation to both 'organizations' and 'institutions' can be seen in the notion of structures of governance. Using the definition provided by the *Oxford Handbook of Governance* as 'the architecture of formal and informal institutions', structures of governance can be seen as comprised of components such as 'systems of rules', 'regimes of laws, rules, judicial decisions, and administrative practices', and 'the comparatively stable institutional, socio-economic and ideational parameters as well as the historically entrenched actor constellations' (Levi-Faur 2012). Used in relation to structured organizations such as the WHO (Carney and Bennett 2014), frameworks such as the IHR (Collins *et al.* 2021), or the structures of government hierarchies within national jurisdictions (Liu, Zhong and Yu 2020; Wang *et al.* 2021; Finnane), this conception of governance is similar to the ways in which 'organizations' and 'institutions' have been used to characterize the same subject area, and underscores the fluidity in which the four terms of institutions, politics, organizations, and governance have been used so far. It may be worth noting that the variety of outcomes experienced in countries with very different organizational and governance structures and processes might argue for some caution in applying these values-based assessments in relation to epidemic and pandemic response.

Additional intersections between conceptions of 'governance' and 'institutions', and even the use of terms to define each other, can be seen in descriptions of 'governance' as a mechanism and as a process, categorizations both also borrowed from the *Oxford Handbook of Governance.* The former, described as being involved with the 'institutional procedures of decision-making'[4], seeks to understand better the 'naturalization' of decision making, through mechanisms such as exchanges – monetized or not –, commands in a hierarchy, persuasion and deliberation, as well as group identity and loyalty (Levi-Faur 2012).

The conception of governance as a process builds upon the relatively stable components of structures and adds 'dynamic interactive aspects' such as steering and coordinating the 'practices of governing' or the 'exercise of authority' in policy-making, through which norms of governance are processed as well (Levi-Faur 2012). Here too, is significant overlap with definitions of governance and institutions, particularly in regards to definitions of the latter which invoke the 'institutionalization' of norms within a jurisdiction or acting organization.

Another way in which this prefix tendency arose was the use of the terms themselves as a prefix in a broader phrase, such as 'institutional rearrangement', 'institutional resilience' (McCormick and Whitney 2013; Suetgiin Soon, Chou, and Shih-Jiunn Shi 2021; De Angelis and

[4] Here, it appears that the term 'institutions' is being used to refer to 'organizations' in the *Oxford Handbook*, compared with the use of 'institutions' to instead refer to norms, also in the same text.

de Oliveira), 'political systems', or 'political participation' (Burkle 2020). As seen here, in addition to application in regards to the term 'governance', the tendency of applying prefixes to the term has also taken place frequently with 'politics', both in regards to values (like 'good politics') and descriptions (such as 'emergency politics') (Afsahi *et al.* 2020; Lee 2020; Ragozina 2020).

*Implications of this conceptual mapping scoping study for institutions, politics, organizations, and governance research*

This conceptual mapping scoping study has found a substantial body of research reflecting the importance of IPOG factors as factors affecting responses to public health needs and crises. These terms have been defined by authors in the field, albeit often used without precise definition in individual publications. Before the impact of IPOG factors on epidemic and pandemic responses can be measured, it must be known what these terms mean among researchers in the first place.

This body of literature has also been expanding rapidly in recent years with the COVID-19 pandemic, suggesting increased importance of these factors in this global crisis. However, there is considerable diversity in how these terms are defined, used, and related to each other by researchers in the field, which engenders a lack of clarity in describing and analyzing phenomena and prescribing action. While some of these trends have been observed in conceptions of governance in health systems more generally (Barbazza and Tello 2014; NTR, AM and PC 2019), it was found through this research that similar patterns of unclear, varied, and competing definitions existed in regards to characterizations of institutions, politics, and organizations as well, within the particular context of epidemic and pandemic responses.

As the COVID-19 pandemic marches into its third year, there is little indication that the production of literature on upstream determinants of governments' response to epidemics and pandemics will substantially subside in the near future. As this volume of literature continues to increase, there will be an increasing need to establish a degree of consistency in the terminology used in order to lessen confusion among researchers, as well as to improve the applicability of new knowledge across a variety of contexts and settings. To this end, a framework such as the IPOG model set forth by the Working Group on Health Systems Response to COVID-19 at the author's institute (Berman *et al.* 2021), mentioned earlier in the methods section, could be useful in the way it establishes boundaries and strict definitions for each term, as well as in the way in which it clearly sets up concepts and processes to describe how they relate to each other. Wider application of frameworks such as the IPOG model could lead to more consistent analysis of the ways in which IPOG has shaped responses to epidemics and pandemics across jurisdictions.

*Limitations*

A key limitation of this research is the limited ability by the reviewers to keep pace with the rate at which literature on government systems' response to infectious disease outbreaks has been published in the past three years. As previously mentioned in the discussion, literature on

COVID-19 has outpaced work published on other diseases (Ioannidis *et al.* 2021). Similar results have been borne out in this review as well, where included literature on COVID-19 exceeded works on all other diseases combined. We have sought to account for the recent frequency of publication on this topic by conducting an updated search with identical keywords and an updated timeframe which covers the time from which the initial search was conducted in late July 2021 to the time of submission in April 2022. Results from this updated search have since been included in this study. The previously mentioned functional limitations on some of the databases which limited searches on studies published prior too July 2011 to PAIS and Ovid Medline are worth mentioning here as well.

Furthermore, even though our search was inclusive of grey literature, a systematized search directed towards grey literature specifically was not undertaken. Thus, it is likely that a wide variety of grey literature relevant to IPOG factors, from sources such as governments, policy briefs, and organizational documents, were not indexed in the searched databases, and thus missed without a systematic search for grey literature unto itself. Similar difficulties may have been encountered with book chapters and edited volumes, which can be difficult to index in databases. Even with this limitation, some grey literature sources have been included in this research, and the databases used in the searches were chosen with the intention of capturing a wide variety of literature from a multitude of sources.

To assist with searching, the terms “epidemic” and “pandemic” were both used concurrently with each other. Although beyond the scope of the present study, further research could more thoroughly explore the extent to which IPOG terms are used or conceptualized differently in the event of epidemics or pandemics. Reviewers also noted interest in exploring how this growing literature might be analyzed by sub-themes as categories within the IPOG factors as well as how multiple IPOG factors might be referenced together in some literature. We hope this work will stimulate further investigation of this type.

Finally, by restricting our search to the English language, we may also have excluded relevant literature from low- and middle-income countries (e.g. French articles from Western African countries; Spanish or Portuguese articles from Latin American countries).

*Conclusion: Looking forward*

The results from this research have shown that even as interest in the impact of IPOG factors has increased sharply in the past three years during the course of the COVID-19 pandemic, inconsistency and disputes continue to characterize the use and definitions of such terms, generating confusion. The results of this study confirm prior findings to this effect on the state of definitions of governance and in health systems more generally (Levi-Faur 2012; Barbazza and Tello 2014; NTR, AM and PC 2019); this study extends such findings towards writing on the other three terms, and in the context of epidemic and pandemic response as well.

Already, retrospectives on the COVID-19 pandemic have underscored the need among public health researchers and practitioners to better understand the interactions between public health measures and conceptions of politics (Greer *et al.* 2021) and governance (Tam 2021a) in order to ensure the science behind public health measures is effectively used during the next pandemic (Frieden, Buissonnière and McClelland 2021). For example, the Public Health Agency of Canada, in its 'Vision to Transform Canada's Public Health System' (Tam 2021a), has identified 'effective governance across jurisdictions and sectors' as not only a principle element of a 'world-class public health system' (Tam 2021b), but also as a research priority going forward (Tam 2021c). Interest in IPOG terms has also been demonstrated prior to the COVID-19 pandemic through organs such as the United Nations, which includes "strong institutions" as part of its Sustainable Development Goals (Goal 16: Promote just, peaceful and inclusive societies).

The dramatic demands placed on public health organizations during the COVID-19 pandemic has also illustrated the wide variety of organizational structures existing in different jurisdictions. This includes the spectrum of more and less centralized structures in both unitary and federal states. It also includes the role of medical care funders and providers in relation to organizations charged with a greater population health focus and the locus and structure of national institutions of public health such as centers for disease control.

This review was performed to support a wider program of research, which in our conceptualization focuses more attention on decision-making processes (governance) at the locus between political and organizational factors and influenced by broader contextual factors such as institutions and other contextual factors. We hope that this focus enriches our understanding of how IPOG determinants affect system-responses to public health crises. Learning more about these processes may also contribute to better system designs and the laws and regulations which define them.

As the world reflects on its recent and ongoing experiences with COVID-19, there is growing awareness of the importance of the 'upstream' factors discussed in this research. For research to contribute to achieving better outcomes in future crises more investigations of these factors may be needed. Increased attention will also benefit from greater clarity about what is being studied. More explicit definitions of IPOG in terms of their distinct concepts and properties and their interactions in the context of epidemic and pandemic response would be a step in the right direction.

## References

Adeel AB, Catalano M, Catalano O *et al.* COVID-19 Policy Response and the Rise of the Sub-National Governments. *Canadian Public Policy* 2020;**46**:565.

Afsahi A, Beausoleil E, Dean R *et al.* Democracy in a Global Emergency Five Lessons from the COVID-19 Pandemic. *DEMOCRATIC THEORY-AN INTERDISCIPLINARY JOURNAL* 2020;**7**:V–XIX.

Agartan TI, Cook S, Lin V. Introduction: COVID-19 and WHO: Global institutions in the context of shifting multilateral and regional dynamics. *Global Social Policy* 2020;**20**:367–73.

Anderson S, Allen P, Peckham S *et al.* Asking the right questions: Scoping studies in the commissioning of research on the organisation and delivery of health services. *Health Research Policy and Systems* 2008;**6**.

Barbazza E, Tello J. A review of health governance: Definitions, dimensions and tools to govern. *Health Policy* 2014;**116**:1–11.

Bardosh KL, Scoones JC, Grace D *et al.* Engaging research with policy and action: what are the challenges of responding to zoonotic disease in Africa? *Philosophical Transactions: Biological Sciences* 2017;**372**:1–10.

Béland D, Cantillon B, Hick R *et al.* Social policy in the face of a global pandemic: Policy responses to the COVID‐19 crisis. *Social Policy and Administration* 2021;**55**:249–60.

Berman P, Keidar S, Zahid M *et al.* Same disease, similar measures, varied outcomes: Research to improve understanding of why results in curbing COVID-19 has been so different across jurisdictions around the world? *University of British Columbia Medical Journal* 2021;**13**:7–8.

Biehl J, Prates LEA, Amon JJ. Supreme Court v. Necropolitics: The Chaotic Judicialization of COVID-19 in Brazil. *Health and Human Rights* 2021;**23**:151.

Boyce MR, Katz R. COVID-19 and the proliferation of urban networks for health security. *Health Policy and Planning* 2021;**36**:357–9.

Brahmbhatt M, Jonas O. International Cooperative Responses to Pandemic Threats: A Critical Analysis. *The Brown Journal of World Affairs* 2015;**21**:164–79.

Brandt A, Gardner M. Antagonism and Accommodation: Interpreting the Relationship Between Public Health and Medicine in the United States During the 20th Century. *AJPH* 2000;**90**:707–15.

Braun V, Clarke V. Using thematic analysis in psychology. *Qualitative Research in Psychology* 2006;**3**:77–101.

Brubacher LJ, Hasan MZ, Sriram V *et al.* Investigating the influence of institutions, politics, organizations, and governance on the COVID-19 response in British Columbia, Canada: A jurisdictional case study protocol. 2022.

Burkle FM. Declining Public Health Protections within Autocratic Regimes: Impact on Global Public Health Security, Infectious Disease Outbreaks, Epidemics, and Pandemics. *Prehospital Disaster Med* 2020;**35**:237–46.

Calain P, Abu Sa'Da C. Coincident polio and Ebola crises expose similar fault lines in the current global health regime. *Confl Health* 2015;**9**:29.

Carney T, Bennett B. Framing pandemic management: New governance, science or culture? *HEALTH SOCIOLOGY REVIEW* 2014;**23**:136–47.

Choi H, Kim S-Y, Kim J-W *et al.* Mainstreaming of Health Equity in Infectious Disease Control Policy During the COVID-19 Pandemic Era. *J Prev Med Pub Health* 2021;**54**:1–7.

Christensen T, Lægreid P. Balancing governance capacity and legitimacy - how the Norwegian government handled the COVID-19 crisis as a high performer. *Public administration review* 2020:10.1111/puar.13241.

Coco A, Dias T. Prevent, Respond, Cooperate States' Due Diligence Duties vis-a-vis the covid-19 Pandemic. *JOURNAL OF INTERNATIONAL HUMANITARIAN LEGAL STUDIES* 2020;**11**:218–36.

Collins T, Tello J, Van Hilten M *et al.* Addressing the double burden of the COVID-19 and noncommunicable disease pandemics: a new global governance challenge. *INTERNATIONAL JOURNAL OF HEALTH GOVERNANCE* 2021;**26**:199–212.

Craig P, Ruggerio ED, Frohlich K *et al. Taking Account of Context in Population Health Intervention Research: Guidance for Producers, Users and Funders of Research.* Southampton: Canadian Institutes of Health Research-National Institute for Health Research, 2018.

De Angelis G, de Oliveira E. COVID-19 and the "state of exception": assessing institutional resilience in consolidated democracies - a comparative analysis of Italy and Portugal. *DEMOCRATIZATION*, DOI: 10.1080/13510347.2021.1949296.

Dostal JM. Governing Under Pressure: German Policy Making During the Coronavirus Crisis. *Polit Q* 2020, DOI: 10.1111/1467-923X.12865.

Doyle JS. An international public health crisis: can global institutions respond effectively to HIV/AIDS? *Australian Journal of International Affairs* 2006;**60**:400–11.

Everts J. Announcing Swine Flu and the Interpretation of Pandemic Anxiety. *Antipode* 2013;**45**:809–25.

Fairchild A, Rosner D, Colgrove J *et al.* The Exodus of Public Health: What History Can Tell Us About the Future. *AJPH* 2010;**100**:54–63.

Fei D. Assembling Chinese health engagement in Africa: structures, strategies and emerging patterns. *THIRD WORLD QUARTERLY*, DOI: 10.1080/01436597.2022.2042802.

Finnane M. Governing in a Pandemic: Law and Government in Australia, 1919. *AUSTRALIAN HISTORICAL STUDIES*, DOI: 10.1080/1031461X.2021.1920990.

Forestier C, Cox AT, Horne S. Coordination and relationships between organisations during the civil-military international response against Ebola in Sierra Leone: an observational discussion. *J R Army Med Corps* 2016;**162**:156–62.

Forum on Medical and Public Health Preparedness for Catastrophic Events, Forum on Drug Discovery D and Translation, Forum on Microbial Threats *et al.* 2016, DOI: 10.17226/21809.

Foucault M. Chapter Four: Governmentality. *The Foucault Effect: Studies in Governmentality, with Two Lectures by and an Interview with Michel Foucault*. Chicago: The University of Chicago Press, 1991, 87–104.

Fowler L, Kettler J, Witt S. Pandemics and Partisanship: Following Old Paths into Uncharted Territory. *AMERICAN POLITICS RESEARCH* 2021;**49**:3–16.

Frieden T, Buissonnière M, McClelland A. The world must prepare now for the next pandemic. *BMJ Global Health* 2021;**6**, DOI: 10.1136/bmjgh-2021-005184.

GBD 2019 Diseases and Injuries Collaborators. Global burden of 369 diseases and injuries in 204 countries and territories, 1990–2019: a systematic analysis for the Global Burden of Disease Study 2019. *The Lancet* 2020;**396**:1204–22.

Gerwin LE. Planning for pandemic: a new model for governing public health emergencies. *Am J Law Med* 2011;**37**:128–71.

Goal 16: Promote just, peaceful and inclusive societies. *United Nations*.

Gostin LO, Katz R. The International Health Regulations: The Governing Framework for Global Health Security. *The Milbank Quarterly* 2016;**94**:264–313.

Greer SL, King EJ, Massard da Fonseca E *et al. Coronavirus Politics: The Comparative Politics and Policy of COVID-19*. Ann Arbor: University of Michigan Press, 2021.

Greer SL PhD, Singer PM MHSA. Addressing Zika in the United States: Polarization, Fragmentation, and Public Health. *American Journal of Public Health* 2017;**107**:861–2.

Hanrieder T, Kreuder-Sonnen C. WHO decides on the exception? Securitization and emergency governance in global health. *Security Dialogue* 2014;**45**:331–48.

He A, Shi Y, Liu H. Crisis governance, Chinese style: distinctive features of China's response to the Covid-19 pandemic. *POLICY DESIGN AND PRACTICE* 2020;**3**:242–58.

Hier S. Narrating the crisis: Moral regulation, overlapping responsibilities and COVID-19 in Canada. *CURRENT SOCIOLOGY*, DOI: 10.1177/00113921211055860.

Hirsch JS, Giang LM, Parker RG *et al.* Caught in the Middle: The Contested Politics of HIV/AIDS and Health Policy in Vietnam. *Journal of Health Politics, Policy and Law* 2015;**40**:13.

Hoffman SJ. The evolution, etiology and eventualities of the global health security regime. *Health Policy and Planning* 2010;**25**:510–22.

Honigsbaum M. Between Securitisation and Neglect: Managing Ebola at the Borders of Global Health. *Med Hist* 2017;**61**:270–94.

Huang H, Peng Z, Wu H *et al.* A big data analysis on the five dimensions of emergency management information in the early stage of COVID-19 in China. *JOURNAL OF CHINESE GOVERNANCE* 2020;**5**:213–33.

Huang K. Minimizing the Social Dilemma. *Investigación Económica* 2021;**80**:32–55.

Ioannidis J, Salholz-Hillel M, Boyack K *et al.* The rapid, massive growth of COVID-19 authors in the scientific literature. *Royal Society Open Science* 2021;**8**.

*Jacobellis v. Ohio*., 1964.

Jones E, MacDougall H, Monnais L *et al. Beyond the COVID-19 Crisis: Building on Lost Opportunities in the History of Public Health*. Ottawa: Royal Society of Canada, 2021.

Jönsson C, Jönsson K. Global and Local Health Governance: Civil society, human rights and HIV/AIDS. *Third World Quarterly* 2012;**33**:1719–34.

Kamradt-Scott A. THE POLITICS OF MEDICINE AND THE GLOBAL GOVERNANCE OF PANDEMIC INFLUENZA. *International Journal of Health Services* 2013;**43**:105–21.

Kim M-H, Cho W, Choi H *et al.* Assessing the South Korean Model of Emergency Management during the COVID-19 Pandemic. *ASIAN STUDIES REVIEW* 2020;**44**:567–78.

Kim Y, Oh S, Wang C. From Uncoordinated Patchworks to a Coordinated System: MERS-CoV to COVID-19 in Korea. *AMERICAN REVIEW OF PUBLIC ADMINISTRATION* 2020;**50**:736–42.

Kim YS. World Health Organization and Early Global Response to HIV/AIDS: Emergence and Development of International Norms. *Journal of International and Area Studies* 2015;**22**:19–40.

Lawson J, Xu F. SARS in Canada and China: Two Approaches to Emergency Health Policy. *Governance: An International Journal of Policy, Administration, and Institutions* 2007;**20**:209–32.

Lee K. WHO under fire: The need to elevate the quality of politics in global health. *Global Social Policy* 2020;**20**:374–7.

Levi-Faur D. From “Big Government” to “Big Governance”? *The Oxford Handbook of Governance*. 2012.

Liesen L, Walsh B. The competing meanings of “biopolitics” in political science: Biological and postmodern approaches to politics. *Politics and the Life Sciences* 2015;**31**:2–15.

Liu P, Zhong X, Yu S. Striking a balance between science and politics: understanding the risk-based policy-making process during the outbreak of COVID-19 epidemic in China. *JOURNAL OF CHINESE GOVERNANCE* 2020;**5**:198–212.

Liu Y. China’s public health-care system: facing the challenges. *World Health Organization Bulletin of the World Health Organization* 2004;**82**:532–8.

Liu Z, Guo J, Zhong W *et al.* Multi-Level Governance, Policy Coordination and Subnational Responses to COVID-19: Comparing China and the US. *Journal of Comparative Policy Analysis: Research and Practice* 2021;**23**:204–18.

Makarychev A, Romashko T. Precarious Sovereignty in a Post-liberal Europe: The COVID-19 Emergency in Estonia and Finland. *CHINESE POLITICAL SCIENCE REVIEW* 2021;**6**:63–85.

McCormick S, Whitney K. The making of public health emergencies: West Nile virus in New York City. *SOCIOLOGY OF HEALTH & ILLNESS* 2013;**35**:268–79.

Means A. Foucault, biopolitics, and the critique of state reason. *Educational Philosophy and Theory* 2021.

Meszaros G. Carl Schmitt in Hungary: Constitutional Crisis in the Shadow of Covid-19. *REVIEW OF CENTRAL AND EAST EUROPEAN LAW* 2021;**46**:69–90.

Moulds S. Scrutinising COVID-19 laws: An early glimpse into the scrutiny work of federal parliamentary committees. *Alternative Law Journal* 2020;**45**:180.

Nelson MJ. Pandemic Politics in South Asia: Muslims and Democracy. *The Review of Faith & International Affairs* 2021;**19**:83–94.

Nguyen VK. Government-by-exception: Enrolment and experimentality in mass HIV treatment programmes in Africa. *Social Theory & Health* 2009;**7**:196–217.

Nohrstedt D, Baekkeskov E. Political drivers of epidemic response: foreign healthcare workers and the 2014 Ebola outbreak. *Disasters* 2018;**42**:41–61.

NTR A, AM R, PC L. The Literature Review of the Governance Frameworks in Health System. *Journal of Public Administration and Governance* 2019;**9**:252–60.

Peters MDJ, Godfrey CM, Khalil H *et al.* Guidance for conducting systematic scoping reviews. *International Journal of Evidence-Based Healthcare* 2015;**13**:141–6.

Ragozina S. Islamic Biopolitics during Pandemics in Russia Intertextuality of Religious, Medical and Political Discourses. *ANTHROPOLOGY IN ACTION-JOURNAL FOR APPLIED ANTHROPOLOGY IN POLICY AND PRACTICE* 2020;**27**:82–6.

Rocco P, Beland D, Waddan A. Stuck in neutral? Federalism, policy instruments, and counter-cyclical responses to COVID-19 in the United States. *POLICY AND SOCIETY* 2020;**39**:458–77.

Rosella L, Wilson K, Crowcroft N *et al.* Pandemic H1N1 in Canada and the use of evidence in developing public health policies - A policy analysis. *SOCIAL SCIENCE & MEDICINE* 2013;**83**:1–9.

Sandset T. The necropolitics of COVID-19: Race, class and slow death in an ongoing pandemic. *Global Public Health* 2021;**16**:1411–23.

Sanyaolu A, Okorie C, Hosein Z *et al.* Global Pandemicity of COVID-19: Situation Report as of June 9, 2020. *Infectious Diseases: Research and Treatment* 2021;**14**, DOI: https://doi.org/10.1177/1178633721991260.

Schwartz J, Evans RG, Greenberg S. Evolution of Health Provision in Pre-SARS China: The Changing Nature of Disease Prevention. *China Review* 2007;**7**:81–104.

Shultz L, Melody V. What are we saving? Tracing governing knowledge and truth discourse in global COVID-19 policy responses. *International Review of Education* 2021;**67**:219–39.

Speakman EM, Burris S, Coker R. Pandemic legislation in the European Union: Fit for purpose? The need for a systematic comparison of national laws. *Health Policy* 2017;**121**:1021.

Suetgiin Soon, Chou CC, Shih‐Jiunn Shi. Withstanding the plague: Institutional resilience of the East Asian welfare state. *Social Policy and Administration* 2021;**55**:374–87.

Tam T. *The Chief Public Health Officer of Canada's Report on the State of Public Health in Canada 2021: A Vision to Transform Canada's Public Health System*. Ottawa: Public Health Agency of Canada, 2021a:129.

Tam T. *Report Summary: A Vision to Transform Canada's Public Health System*. Ottawa: Public Health Agency of Canada, 2021b:12.

Tam T. *Generating Knowledge to Inform Public Health System Transformation*. Ottawa: Public Health Agency of Canada, 2021c:7.

Teigen PM. Legislating fear and the public health in gilded age Massachusetts. *Journal of the history of medicine and allied sciences* 2007;**62**:141–70.

Thomson S, Ip EC. COVID-19 emergency measures and the impending authoritarian pandemic. *J Law Biosci* 2020;**7**:lsaa064.

Tricco AC, Lillie E, Zarin W *et al.* PRISMA extension for scoping reviews (PRISMA-ScR): checklist and explanation. *Annals of Internal Medicine* 2018;**169**:467–73.

US Department of Health and Human Services. What is the U.S. Opioid Epidemic? 2021.

Vankovska B. Dealing with COVID-19 in the European periphery: between securitization and "gaslighting." *Journal of Global Faultlines* 2020;**7**:71–88.

Wang C, Dong X, Zhang Y *et al.* Community Resilience Governance on Public Health Crisis in China. *INTERNATIONAL JOURNAL OF ENVIRONMENTAL RESEARCH AND PUBLIC HEALTH* 2021;**18**, DOI: 10.3390/ijerph18042123.

Wenham C. Regionalizing Health Security. *Contemporary Southeast Asia* 2018;**40**:126–51.

West-Oram P. Solidarity is for other people: identifying derelictions of solidarity in responses to COVID-19. *JOURNAL OF MEDICAL ETHICS* 2021;**47**:65–8.

Wilson K, Lazar H. From SARS to Avian flu--why Ottawa must lead Canada's response. *Policy Options/Options Politiques* 2006;**27**:31–8.

Windholz E. Governing in a pandemic: from parliamentary sovereignty to autocratic technocracy. *THEORY AND PRACTICE OF LEGISLATION* 2020;**8**:93–113.

Wu A. Review of the book Coronavirus Politics: The Comparative Politics and Policy of COVID-19, by S. Greer et al. *WMHP* 2022, DOI: 10.1002/wmh3.505.

Yan B, Chen B, Wu L *et al.* Culture, Institution, and COVID-19 First-Response Policy: A Qualitative Comparative Analysis of Thirty-One Countries. *Journal of Comparative Policy Analysis: Research and Practice* 2021;**23**:219–33.

Yao D, Li J, Chen Y *et al.* Interactive Governance Between and Within Governmental Levels and Functions: A Social Network Analysis of China's Case Against COVID-19. *AMERICAN REVIEW OF PUBLIC ADMINISTRATION*, DOI: 10.1177/02750740211059534.

Zhang J, Zhang R. COVID-19 in China: Power, Transparency and Governance in Public Health Crisis. *HEALTHCARE* 2020;**8**, DOI: 10.3390/healthcare8030288.

Zhang X, Wang L. Administrative Governance and Frontline Officers in the Chinese Prison System During the COVID-19 Pandemic. *Asian J Criminol* 2021:1–17.

Zhou S, Han L, Liu P *et al.* Global health governance for travel health: lessons learned from the coronavirus disease 2019 (COVID-19) outbreaks in large cruise ships. *Glob health j* 2020;**4**:133–8.

***Figure 1.*** Diagram depicting the flow if identification, screening, and eligibility assessment of articles included in this study.

ACCEPTED

***Figure 2.*** Summary of the number of publications per year (2004-2022) related to IPOG factors affecting epidemic and pandemic response.

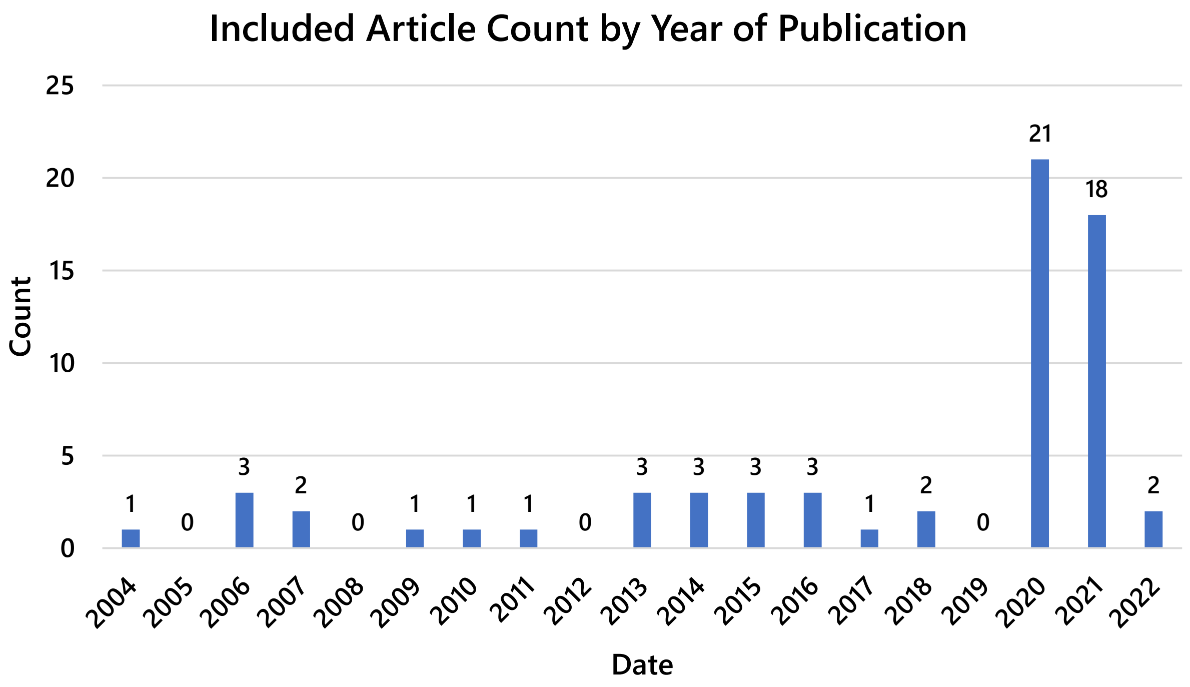

ACCEPTED MA

***Figure 3.*** Article counts by disease studied. Note that totals exceed 65 and proportions exceed 100%, as some articles discussed multiple diseases.

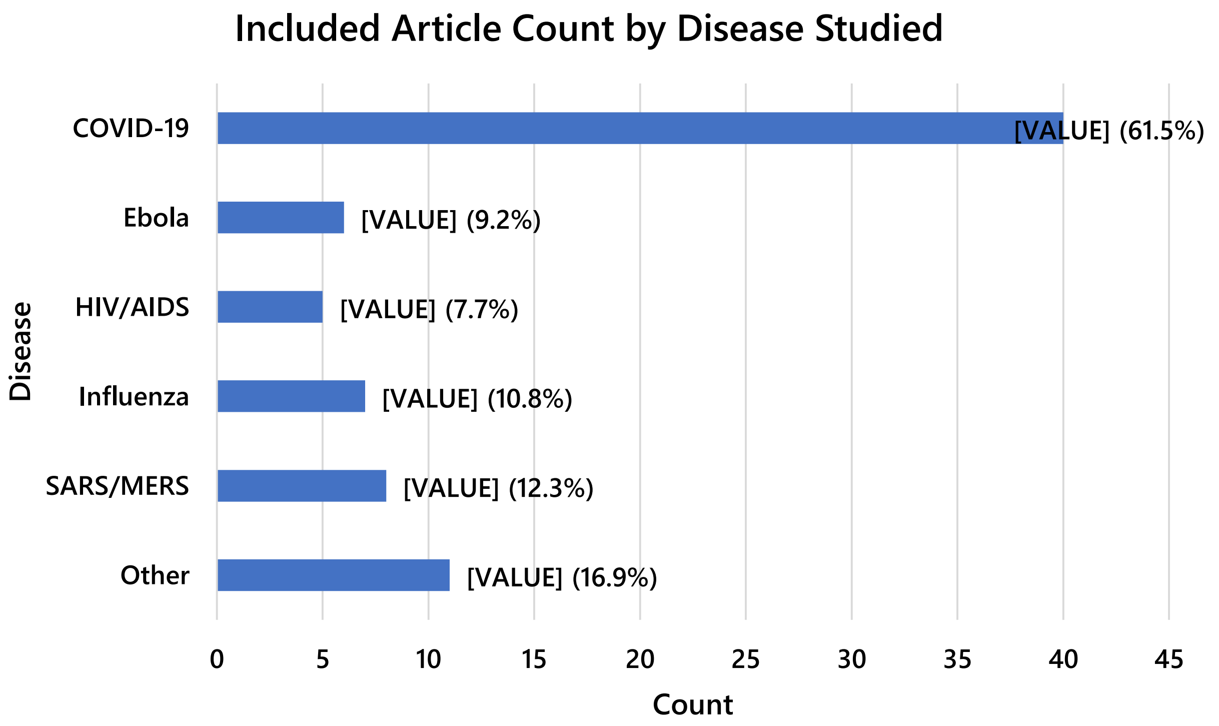

ACCEPTED MA

***Table 1.*** Search string used in Web of Science™ and Ovid Medline® databases and adapted to other databases to retrieve relevant records for this study (see Appendix B for a comprehensive list of search strings used in each database).

| **Category** | **Terms** |
|---|---|
| ‘IPOG’ Terms | (Institution* OR legal OR enforc* OR organi?ation OR politc* OR ideolog* OR elect* OR policy OR decision OR sociopolitical OR govern* OR regulation* OR regulatory)<br><br>**AND** |
| Health Crisis Terms | (Pandemic* OR ‘infectious disease event’ OR epidemic*)<br>**AND**<br>(‘state of emergenc*’ OR ‘declaration adj4 emergenc*’ OR ‘public health crisis’ OR ‘public health emergenc*’ OR ‘state adj4 emergenc*’)<br>**NOT**<br>(opioid OR opinion OR pre-pandemic OR survey) |

***Table 2***. List of included articles retrieved from a systematic search of the published academic literature on IPOG factors in epidemic and pandemic response. Articles are organized chronologically by year of publication, with author(s) title, study region(s), and factor(s) studied also indicated.

| **Year** | **Author(s)** | **Title** | Region(s)[1] | **Term(s) Defined** |
|---|---|---|---|---|
| 2006 | Policy Options (digital magazine) | From SARS to Avian flu--why Ottawa must lead Canada's response | Americas | Governance |
| 2009 | Nguyen VK. | Government-by-exception: Enrolment and experimentality in mass HIV treatment programmes in Africa | Africa; Multilateral | Governance |
| 2010 | Hoffman SJ. | The evolution, etiology and eventualities of the global health security regime | Multilateral | Governance |
| 2011 | Gerwin LE. | Planning for pandemic: a new model for governing public health emergencies. | Americas | Governance |
| 2013 | Scott AK. | THE POLITICS OF MEDICINE AND THE GLOBAL GOVERNANCE OF PANDEMIC INFLUENZA | Multilateral | Governance |
| 2014 | Hanrieder T, Sonnen CK. | WHO decides on the exception? Securitization and emergency governance in global health | Multilateral | Governance |
| 2014 | Carney T, Bennet B. | Framing pandemic management: New governance, science or culture? | Oceania; Multilateral | Governance |

[1] Regions are based upon the UN Geoscheme, visualized in Figure 4; 'multilateral' refers to articles which studied entities with no geographic borders, such as the UN, WHO, or World Bank.

| | | | | |
|---|---|---|---|---|
| 2014 | Roodenrijs JCM, Kraaij-Dirkzwager M.M, Kerkhof J.H.T.C vd et al. | Risk governance for infectious diseases: exploring the feasibility and added value of the IRGC-framework for Dutch infectious disease control | Europe | Governance |
| 2015 | Calain P, Sa'Da CA. | Coincident polio and Ebola crises expose similar fault lines in the current global health regime | Africa; Multilateral | Governance |
| 2015 | Hirsch JS, Giang LM, Parker RG et al. | Caught in the Middle: The Contested Politics of HIV/AIDS and Health Policy in Vietnam | Asia | Governance |
| 2016 | Mack A, Snair MR, Choffnes ER. | Global Health Risk Framework: Governance for Global Health Workshop Summary | Multilateral | Governance |
| 2016 | Gostin L, Katz R. | The International Health Regulations: The Governing Framework for Global Health Security | Multilateral | Governance |
| 2017 | Speakman EM, Burris S, Coker R. | Pandemic legislation in the European Union: Fit for purpose? The need for a systematic comparison of national laws | Europe; Multilateral | Governance |
| 2018 | Wenham C. | Regionalizing Health Security | Asia; Multilateral | Governance |
| 2020 | Zhang J, Zhang R. | COVID-19 in China: Power, Transparency and Governance in Public Health Crisis | Asia | Governance |
| 2020 | Kim MH, Cho W, Choi H et al. | Assessing the South Korean Model of Emergency Management during the COVID-19 Pandemic | Asia | Governance |
| 2020 | Christensen t, Lægreid P. | Balancing governance capacity and legitimacy - how the Norwegian government handled the COVID-19 crisis as a high performer | Europe | Governance |
| 2020 | Collins T, Tello J, Hilten MV et al | Addressing the double burden of the COVID-19 and noncommunicable disease pandemics: a new global governance challenge | Multilateral | Governance |
| 2020 | Agartan TI, Cook S, Lin V | Introduction: COVID-19 and WHO: Global institutions in the context of shifting multilateral and regional dynamics | Multilateral | Governance |
| 2020 | Coco A, Dias Td S. | Prevent, Respond, Cooperate States' Due Diligence Duties vis-a-vis the covid-19 Pandemic | Multilateral | Governance |
| 2020 | Zhoua S, Hana L, Liua P et al. | Global health governance for travel health: lessons learned from the coronavirus disease 2019 (COVID-19) outbreaks in large cruise ships. | Multilateral | Governance |
| 2021 | Finnane M. | Governing in a Pandemic: Law and Government in Australia, 1919 | Oceania | Governance |
| 2021 | Areal AG, Sheppy B. | A Crisis of Governance – Or an Opportunity? | Europe | Governance |
| 2021 | Thomson S, Ip EC. | COVID-19 emergency measures and the impending authoritarian pandemic. | Africa; Americas; Asia; Europe; Oceania; Multilateral | Governance |
| 2021 | Mészáros G. | Carl Schmitt in Hungary: Constitutional Crisis in the Shadow of Covid-19 | Europe | Governance |
| 2021 | Shultz L, Viczko M. | What are we saving? Tracing governing knowledge and truth discourse in global COVID-19 policy responses | Multilateral | Governance |

| | | | | |
|---|---|---|---|---|
| 2021 | Wang C, Dong X, Zhang Y et al. | Community Resilience Governance on Public Health Crisis in China | Asia | Governance |
| 2021 | Zhang X, Wang L. | Administrative Governance and Frontline Officers in the Chinese Prison System During the COVID-19 Pandemic. | Asia | Governance |
| 2022 | Yao D, Li J, Chen Y et al. | Interactive Governance Between and Within Governmental Levels and Functions: A Social Network Analysis of China's Case Against COVID-19 | Asia | Governance |
| 2020 | Windholz EL. | Governing in a pandemic: from parliamentary sovereignty to autocratic technocracy | Oceania | Institutions; Governance |
| 2020 | He AJ, Shi Y, Liu H. | Crisis governance, Chinese style: distinctive features of China's response to the Covid-19 pandemic | Asia | Institutions; Governance |
| 2021 | Goetz KH, Martinsen DS. | COVID-19: a dual challenge to European liberal democracy | Europe | Institutions; Governance |
| 2006 | Doyle JS. | An international public health crisis: can global institutions respond effectively to HIV/AIDS? | Multilateral | Institutions |
| 2013 | McCormick S, Whitney K. | The making of public health emergencies: West Nile virus in New York City | Americas | Institutions |
| 2013 | Rosella LC, Wilson K, Crowcroft NS et al. | Pandemic H1N1 in Canada and the use of evidence in developing public health policies - A policy analysis | Americas | Institutions |
| 2015 | Kim YS. | World Health Organization and Early Global Response to HIV/AIDS: Emergence and Development of International Norms | Multilateral | Institutions |
| 2018 | Nohrstedt D, Baekkeskov E. | Political drivers of epidemic response: foreign healthcare workers and the 2014 Ebola outbreak | Africa | Institutions |
| 2021 | Angelis GD, Oliveira Ed. | COVID-19 and the "state of exception": assessing institutional resilience in consolidated democracies - a comparative analysis of Italy and Portugal | Europe | Institutions |
| 2021 | West-Oram P. | Solidarity is for other people: identifying derelictions of solidarity in responses to COVID-19 | Europe | Institutions |
| 2021 | Yan B, Chen B, Wu L, Zhang X et al. | Culture, Institution, and COVID-19 First-Response Policy: A Qualitative Comparative Analysis of Thirty-One Countries | Asia; Europe; Oceania; Multilateral | Institutions |
| 2021 | Soon S, Chou C, Shi SJ. | Withstanding the plague: Institutional resilience of the East Asian welfare state | Asia | Institutions |
| 2022 | Fei D. | Assembling Chinese health engagement in Africa: structures, strategies and emerging patterns | Africa; Asia | Institutions |
| 2020 | Adeel AB, Catalano M, Catalano O et al. | COVID-19 Policy Response and the Rise of the Sub-National Governments | Americas | Institutions; Politics |
| 2020 | Vankovska B. | Dealing with COVID-19 in the European periphery: between securitization and gaslighting | Europe | Institutions; Politics |
| 2006 | Teigen PM. | Legislating fear and the public health in gilded age Massachusetts. | Americas | Politics |
| 2007 | Lawson J, Feng X. | SARS in Canada and China: Two Approaches to Emergency Health Policy | Americas; Asia | Politics |
| 2020 | Burkle, F.M., Jr. | Declining Public Health Protections within Autocratic Regimes: Impact on Global Public | Africa; Americas; | Politics |

| | | | | |
|---|---|---|---|---|
| | | Health Security, Infectious Disease Outbreaks, Epidemics, and Pandemics | Asia; Europe | |
| 2020 | Ragozina SA. | Islamic Biopolitics during Pandemics in Russia Intertextuality of Religious, Medical and Political Discourses | Europe | Politics |
| 2020 | Dostal JM. | Governing Under Pressure: German Policy Making During the Coronavirus Crisis. | Europe | Politics |
| 2020 | Lee K. | WHO under fire: The need to elevate the quality of politics in global health | Multilateral | Politics |
| 2020 | Afsahi A, Beausoleil E, Dean R et al. | Democracy in a Global Emergency Five Lessons from the COVID-19 Pandemic | Multilateral | Politics |
| 2020 | Moulds S. | Scrutinising COVID-19 laws: An early glimpse into the scrutiny work of federal parliamentary committees | Oceania | Politics |
| 2021 | Sandset T. | The necropolitics of COVID-19: Race, class and slow death in an ongoing pandemic | Europe | Politics |
| 2021 | Nelson MJ. | Pandemic Politics in South Asia: Muslims and Democracy | Asia | Politics |
| 2021 | Biehl J, Prates LEA, Amon JJ. | Supreme Court v. Necropolitics: The Chaotic Judicialization of COVID-19 in Brazil | Americas | Politics |
| 2021 | Fowler L, Kettler J, Witt S. | Pandemics and Partisanship: Following Old Paths into Uncharted Territory | Americas | Politics |
| 2021 | Hier S. | Narrating the crisis: Moral regulation, overlapping responsibilities and | Americas | Politics |
| 2020 | Liu P, Zhong X, Yu S. | Striking a balance between science and politics: understanding the risk-based policy-making process during the outbreak of COVID-19 epidemic in China | Asia | Politics; Governance |
| 2020 | Makarychev A, Romashko T. | Precarious Sovereignty in a Post-liberal Europe: The COVID-19 Emergency in Estonia and Finland | Europe | Politics; Governance |
| 2004 | Liu Y. | China's public health-care system: facing the challenges | Asia | Organizations |
| 2007 | Schwartz J, Evans RG, Greenberg S. | Evolution of Health Provision in Pre-SARS China: The Changing Nature of Disease Prevention | Asia | Organizations |
| 2016 | Forestier C, Cox AT, Horne S. | Coordination and relationships between organisations during the civil-military international response against Ebola in Sierra Leone: an observational discussion | Asia | Organizations |
| 2020 | Boyce MR, Katz R. | COVID-19 and the proliferation of urban networks for health security | Multilateral | Organizations |
| 2020 | Kim Y, Oh SS, Wang C. | From Uncoordinated Patchworks to a Coordinated System: MERS-CoV to COVID-19 in Korea | Asia | Organizations |
| 2021 | Liu Z, Guo J, Zhong W et al. | Multi-Level Governance, Policy Coordination and Subnational Responses to COVID-19: Comparing China and the US | Americas; Asia | Institutions; Organizations |

***Table 3***. Counts and proportions of articles by region of focus. Regions are based upon the UN Geoscheme.

| Region name | Article count | Article proportion[5] |
|---|---|---|
| Africa | 6 | 9.2% |
| Americas | 13 | 20% |
| Asia | 21 | 32.3% |
| Europe | 16 | 24.6% |
| Oceania | 6 | 9.2% |
| Multilateral[6] | 22 | 33.8% |

***Table 4***. Examples of definitions for 'institutions' in the included literature are as follows:

| | |
|---|---|
| **Examples of definitions for 'institutions'** | 1. 'Institutional rearrangement, draws attention to the processes through which the policies and procedures put in place during emergency declarations ramify, and permanently change the face of public health governance.' [29] |
| | 2. 'Factors that create such continuity and predictability are commonly labelled institutions and include rules, norms, widely shared ideas, and other enduring socioeconomic and political structures that mould behaviour' [30] |
| | 3. 'Solidarity is fully institutionalised, 'in the form of legally enforceable norms', such as progressive tax systems and welfare state arrangements' [31] |
| | 4. 'Institutional context refers to the systems and processes that countries use to structure authority, attention, information flows, and relationships in addressing policy problems … institutional factors are concerned with the formal power structure, legal system, and regulations, whereas culture orientation emphasizes the informal norms, beliefs, values, and customs' [32] |

[5] Proportions and counts exceed 100% as some articles examined jurisdictions in multiple regions.
[6] Articles which studied entities with no geographic borders, such as the UN, WHO, or World Bank.

***Table 5***. Examples of definitions for 'governance' in the included literature are as follows:

| | |
|---|---|
| **Examples of definitions** | 1. 'In the context of infectious disease outbreaks of global significance, governance encompasses a range of integrated policy, information management, command, and control mechanisms for facilitating collective action to achieve the objectives of prevention, detection, and response. Of necessity, these mechanisms integrate actions across intergovernmental organizations, sovereign nations, communities, the corporate sector, humanitarian agencies, and civil society. They operate in not only the realm of health, but also to a variable extent in collateral spheres to include agriculture/ food security, diplomacy, education, finance, migration/refugee care, security, and transportation.' (50) |
| | 2. 'The way in which the global health systems are managed' [57] |
| | 3. 'The organized social response to health conditions at the global level.' [57] |
| | 4. Governance capacity as '[The] preparedness or analytical capacity, coordination, regulation and implementation or delivery capacity … to provide effective crisis management', and governance legitimacy as 'citizens' trust in government and concerns such issues as accountability, support, expectations, and reputation' [58] |
| | 5. 'Governance refers to the steering of society with regard to societal problems. Risk governance can be defined as 'both the institutional structure and the policy process that guide and restrain collective activities of a group, society or international community to regulate, reduce or control risk problems'' [59] |
| | 6. Global health governance is defined as 'the use of formal and informal institutions, rules, and processes by states, intergovernmental organizations, and nonstate actors to deal with challenges to health that require cross-border collective action to address |

| | |
|---|---|
| **for 'governance'** | effectively' [60,61] |
| | 7. Adaptive governance is defined as flexible and learning-based multi-level modes of governance or institutional arrangements that can build resilience for the challenges posed by complex and urgent problems [62] |
| | 8. 'Global social governance [is] the mechanisms that enable the international community to address global social problems, through systems of global regulation across national borders and a globally agreed set of social rights' [63] |
| | 9. 'The assignment of authority and the specification of procedures' |
| | 10. '[Multi-level governance] is defined as a governance system within which power is dispersed across government levels vertically and across sectors horizontally' [64] |
| | 11. 'Multi-level governance refers to the institutional arrangements of policy making and implementation that involve continuous interaction and coordination among government and non-government actors across different levels and territories … Type I referring to a system of power sharing among different levels of general-purpose jurisdictions and Type II being essentially a polycentric system of decentralized, overlapping, and competitive jurisdictions' [41] |
| | 12. 'Corporate governance 'is concerned with the structures and systems of control by which managers are held accountable to those that have a legitimate stake in an organisation'' [65] |

**APPENDIX A.** Reporting Items for Systematic reviews and Meta-Analyses extension for Scoping Reviews (PRISMA-ScR) Checklist

| SECTION | ITEM | PRISMA-ScR CHECKLIST ITEM | REPORTED ON PAGE # |
|---|---|---|---|
| **TITLE** | | | |
| Title | 1 | Identify the report as a scoping review. | #1 |
| **ABSTRACT** | | | |
| Structured summary | 2 | Provide a structured summary that includes (as applicable): background, objectives, eligibility criteria, sources of evidence, charting methods, results, and conclusions that relate to the review questions and objectives. | #1 |
| **INTRODUCTION** | | | |
| Rationale | 3 | Describe the rationale for the review in the context of what is already known. Explain why the review questions/objectives lend themselves to a scoping review approach. | #2 |
| Objectives | 4 | Provide an explicit statement of the questions and objectives being addressed with reference to their key elements (e.g., population or participants, concepts, and context) or other relevant key elements used to conceptualize the review questions and/or objectives. | #2 |
| **METHODS** | | | |
| Protocol and registration | 5 | Indicate whether a review protocol exists; state if and where it can be accessed (e.g., a Web address); and if available, provide registration information, including the registration number. | #4 #18 |
| Eligibility criteria | 6 | Specify characteristics of the sources of evidence | #3 #4 |

| SECTION | ITEM | PRISMA-ScR CHECKLIST ITEM | REPORTED ON PAGE # |
|---|---|---|---|
| | | used as eligibility criteria (e.g., years considered, language, and publication status), and provide a rationale. | |
| Information sources* | 7 | Describe all information sources in the search (e.g., databases with dates of coverage and contact with authors to identify additional sources), as well as the date the most recent search was executed. | #3 |
| Search | 8 | Present the full electronic search strategy for at least 1 database, including any limits used, such that it could be repeated. | #3 |
| Selection of sources of evidence† | 9 | State the process for selecting sources of evidence (i.e., screening and eligibility) included in the scoping review. | #4 |
| Data charting process‡ | 10 | Describe the methods of charting data from the included sources of evidence (e.g., calibrated forms or forms that have been tested by the team before their use, and whether data charting was done independently or in duplicate) and any processes for obtaining and confirming data from investigators. | #4 |
| Data items | 11 | List and define all variables for which data were sought and any assumptions and simplifications made. | Appendix (Supplementary Material) |
| Critical appraisal of individual sources of evidence§ | 12 | If done, provide a rationale for conducting a critical appraisal of included sources of evidence; describe the methods used and how this information was used in any data synthesis (if appropriate). | #3 |
| Synthesis of results | 13 | Describe the methods of handling and summarizing the data that were charted. | #3 and #4 |
| **RESULTS** | | | |
| Selection of sources of evidence | 14 | Give numbers of sources of evidence screened, assessed for eligibility, and included in the review, with reasons for exclusions at each stage, ideally using a flow diagram. | #5 |
| Characteristics of sources of evidence | 15 | For each source of evidence, present characteristics for which data were charted and provide the citations. | #5 |
| Critical appraisal within sources of evidence | 16 | If done, present data on critical appraisal of included sources of evidence (see item 12). | #10 and #11 |
| Results of individual sources of evidence | 17 | For each included source of evidence, present the relevant data that were charted that relate to the review questions and objectives. | #5 |
| Synthesis of results | 18 | Summarize and/or present the charting results as they relate to the review questions and objectives. | #5 #6 #7 #8 #9 |
| **DISCUSSION** | | | |
| Summary of evidence | 19 | Summarize the main results (including an overview of concepts, themes, and types of evidence available), link to the review questions and objectives, and consider the relevance to key groups. | #10 |
| Limitations | 20 | Discuss the limitations of the scoping review process. | #15 |
| Conclusions | 21 | Provide a general interpretation of the results with respect to the review questions and objectives, as well as potential implications and/or next steps. | #15 |

| SECTION | ITEM | PRISMA-ScR CHECKLIST ITEM | REPORTED ON PAGE # |
|---|---|---|---|
| **FUNDING** | | | |
| Funding | 22 | Describe sources of funding for the included sources of evidence, as well as sources of funding for the scoping review. Describe the role of the funders of the scoping review. | Title Page #2 |

JBI = Joanna Briggs Institute; PRISMA-ScR = Preferred Reporting Items for Systematic reviews and Meta-Analyses extension for Scoping Reviews.
* Where *sources of evidence* (see second footnote) are compiled from, such as bibliographic databases, social media platforms, and Web sites.
† A more inclusive/heterogeneous term used to account for the different types of evidence or data sources (e.g., quantitative and/or qualitative research, expert opinion, and policy documents) that may be eligible in a scoping review as opposed to only studies. This is not to be confused with *information sources* (see first footnote).
‡ The frameworks by Arksey and O'Malley (6) and Levac and colleagues (7) and the JBI guidance (4, 5) refer to the process of data extraction in a scoping review as data charting.
§ The process of systematically examining research evidence to asses#s its validity, results, and relevance before using it to inform a decision. This term is used for items 12 and 19 instead of "risk of bias" (which is more applicable to systematic reviews of interventions) to include and acknowledge the various sources of evidence that may be used in a scoping review (e.g., quantitative and/or qualitative research, expert opinion, and policy document).

*From:* Tricco AC, Lillie E, Zarin W, O'Brien KK, Colquhoun H, Levac D, et al. PRISMA Extension for Scoping Reviews (PRISMAScR): Checklist and Explanation. Ann Intern Med. 2018;169:467–473. doi: 10.7326/M18-0850

**Appendix B.** Full search strings used in each database, and number of records retrieved.

JSTOR

| Category | Terms |
|---|---|
| IPOG terms | (Institution* OR norm* OR legal OR regulat* OR enforc* OR organi?ation OR politc* OR decision OR ideolog* OR elect* OR power OR policy OR decision OR sociopolitical OR govern*) [Abstract] AND |
| Health Crisis terms | Pandemic [Abstract] |
| Exclusions | N/A |
| Miscellaneous | 'Content I can access', Articles and Research Reports, English |
| Time frame | 26 July 2011 to 26 July 2021<br>26 April 2021 to 8 April 2022 |

PAIS

| Category | Terms |
|---|---|
| IPOG terms | (Institution* OR norm* OR conduct OR legal OR regulat* OR enforc* OR organi?ation OR politic* OR decision OR ideolog* OR official* OR elect* OR power OR policy OR decision OR sociopolitical OR govern*) [Anywhere except full text] AND |
| Health Crisis terms | (Pandemic OR 'infectious disease event' OR epidemic) [Anywhere except full text] |

| | |
|---|---|
| | AND<br>(Emergency OR ‘state of’ OR ‘declaration of’ OR ‘public health crisis’ OR ‘public health emergency’) [Anywhere except full text] |
| Exclusions | NOT (opioid OR opinion OR pre-pandemic OR survey) [Anywhere except full text] |
| Miscellaneous | Source type: Magazines, Reports, and Scholarly Journals<br>Document type: all<br>English |
| Time frame | Before 26 July 2011<br>26 July 2011 to 26 July 2021<br>26 April 2021 to 8 April 2022 |

Web of Science

| Category | Terms |
|---|---|
| IPOG terms | (Institution* OR legal OR enforc* OR organi?ation OR politc* OR ideolog* OR elect* OR policy OR decision OR sociopolitical OR govern* OR regulation* OR regulatory) [Topic] AND |
| Health Crisis terms | (Pandemic* OR ‘infectious disease event’ OR epidemic*) [Topic]<br>AND<br>(‘state of emergenc*’ OR ‘declaration adj4 emergenc*’ OR ‘public health crisis’ OR ‘public health emergenc*’ OR ‘state adj4 emergenc*’) [Topic] |
| Exclusions | NOT (opioid OR opinion OR pre-pandemic OR survey) [Topic] |
| Miscellaneous | English |
| Time frame | 26 July 2011 to 26 July 2021<br>26 April 2021 to 8 April 2022 |

Medline

| Category | Terms |
|---|---|
| IPOG terms | (Institution* OR legal OR enforc* OR organi?ation OR politc* OR ideolog* OR elect* OR policy OR decision OR sociopolitical OR govern* OR regulation* OR regulatory) AND |
| Health Crisis terms | (Pandemic* OR ‘infectious disease event’ OR epidemic*) |

| | |
|---|---|
| | AND<br>(‘state of emergenc*’ OR ‘declaration adj4 emergenc*’ OR ‘public health crisis’ OR ‘public health emergenc*’ OR ‘state adj4 emergenc*’) |
| Exclusions | NOT (opioid OR opinion OR pre-pandemic OR survey) |
| Miscellaneous | English |
| Time frame | 1860 to 2011<br>2011 to 2021<br>2021 to 2022 |

**Appendix C.** More specific inclusion and exclusion criteria for Level 1 screening

| Included | Excluded |
|---|---|
| Articles focused on health/risk communication, e.g. between decision-makers, those in positions of authority, and the general public | Articles on ‘the general public’s perceptions’ of pandemic response or of government action /decisions related to a public health emergency |
| Articles on HIV as related to IPOG factors (considered as a pandemic/public health emergency in this review) | Articles that focus on reporting an emerging public health threat/crisis, which may mention the need for government/organizational action, but not as the focus of the inquiry |
| Articles that are conceptual/theoretical (e.g. not linked to a specific event/emergency response) *if* they are helpful in defining/operationalizing IPOG factors | Articles that are conceptual/theoretical (as a point of inquiry/focus), e.g. using a ‘health and human rights framework’ to conceptualize public health response to emergency; bioethics articles without connection to particular events/people; ‘calls to action’/commentary without analysis of an event/situation |
| Articles on pandemics/epidemics/public health emergencies/spread of infectious illness generally (as related to IPOG & upstream decision-making), including, e.g: pandemic influenza (e.g. H1N1), measles, Severe Acute Respiratory Syndrome (SARS), tuberculosis, Ebola, Zika | Articles on ‘epidemics’ of other kinds, e.g. non-communicable diseases or substance use (e.g. an ‘epidemic’ of childhood obesity or diabetes) |
| Articles that describe the public health and/or government response to an infectious | Articles on bioterrorism/biological weapons as a public health threat (not focused on *responses* but |

| | |
|---|---|
| illness/pandemic/epidemic, including significant reference to IPOG factors (e.g. various organizational structures/roles in the response) | hypothetical threats not yet occurred) |
| Articles on education or training (e.g. of decision-makers in statutory laws/legal mechanisms relevant to pandemic preparedness) in connection to an actual non-communicable disease event/emergency | Articles on vaccine rationing at the 'downstream' level (e.g. among primary healthcare providers, local public health authorities, hospital administrators); vaccine hesitancy/perception among the public; vaccine development (unless focused on policy); or vaccine-injury compensation |
| | Articles focused on research or the place of research in public health governance and emergency response/preparedness |
| | Articles on IPOG as related to mental/behavioural health associated with public health emergencies |
| | Articles on disaster response (not specific to infectious illness/pandemics/epidemics) |
| | Articles on policy/politics surrounding global health aid for pandemics/public health crises |

**Appendix D.** Data extraction questions

- Authors

- Year of publication

- Type of source (multiple-selection checkbox)
  - Primary peer-reviewed article
  - Secondary Review
  - Working paper
  - News item/grey literature
  - Editorial/commentary
  - Quantitative
  - Qualitative
  - Mixed methods
  - Other

- Research question

- Study location (multiple-selection checkbox, based upon UN Geoscheme)
  - Africa
  - Americas
  - Asia
  - Europe
  - Oceania
  - Multilateral (Such as the UN, WHO, World Bank, etc.

- Event(s) studied (multiple-selection checkbox)
  - COVID-19
  - Cholera
  - Ebola
  - HIV/AIDS
  - Influenza
  - SARS/MERS
  - Smallpox
  - Tuberculosis
  - Zika
  - Other (text box entry)

- Disciplinary lens (multiple-selection checkbox)

- Health care
- History
- International relations
- Law
- Philosophy/theory
- Political science
- Public health
- Other (text box entry)

- IPOG terms defined
  - Institutions
  - Politics
  - Organizations
  - Governance
  - Other

- Definitions used of IPOG terms (text box entry)

- Impact of IPOG factors on pandemic response (text box entry)

## Supplementary Material

czac091_Supp

