# How have researchers defined institutions, politics, organizations and governance in research related to epidemic and pandemic response? A scoping review to map current concepts

**DOI:** 10.1093/heapol/czac091

**Published:** 2022-10-29

**Authors:** Austin Wu, Shivangi Khanna, Shelly Keidar, Peter Berman, Laura Jane Brubacher

**Affiliations:** School of Population and Public Health, University of British Columbia, 2206 East Mall, Vancouver, BC V6T 1Z3, Canada; School of Population and Public Health, University of British Columbia, 2206 East Mall, Vancouver, BC V6T 1Z3, Canada; School of Population and Public Health, University of British Columbia, 2206 East Mall, Vancouver, BC V6T 1Z3, Canada; School of Population and Public Health, University of British Columbia, 2206 East Mall, Vancouver, BC V6T 1Z3, Canada; School of Population and Public Health, University of British Columbia, 2206 East Mall, Vancouver, BC V6T 1Z3, Canada

**Keywords:** COVID-19, institutions, politics, organizations, governance, public health, health crisis response, epidemic and pandemic response, preparedness, IPOG definitions

## Abstract

In recent years, the literature on public health interventions and health outcomes in the context of epidemic and pandemic response has grown immensely. However, relatively few of these studies have situated their findings within the institutional, political, organizational and governmental (IPOG) context in which interventions and outcomes exist. This conceptual mapping scoping study synthesized the published literature on the impact of IPOG factors on epidemic and pandemic response and critically examined definitions and uses of the terms IPOG in this literature. This research involved a comprehensive search of four databases across the social, health and biomedical sciences as well as multi-level eligibility screening conducted by two independent reviewers. Data on the temporal, geographic and topical range of studies were extracted, then descriptive statistics were calculated to summarize these data. Hybrid inductive and deductive qualitative analysis of the full-text articles was conducted to critically analyse the definitions and uses of these terms in the literature. The searches retrieved 4918 distinct articles; 65 met the inclusion criteria and were thus reviewed. These articles were published from 2004 to 2022, were mostly written about COVID-19 (61.5%) and most frequently engaged with the concept of governance (36.9%) in relation to epidemic and pandemic response. Emergent themes related to the variable use of the investigated terms, the significant increase in relevant literature published amidst the COVID-19 pandemic, as well as a lack of consistent definitions used across all four terms: institutions, politics, organizations and governance. This study revealed opportunities for health systems researchers to further engage in interdisciplinary work with fields such as law and political science, to become more forthright in defining factors that shape responses to epidemics and pandemics and to develop greater consistency in using these IPOG terms in order to lessen confusion among a rapidly growing body of literature.

Key messages

- Literature on the institutional, political, organizational and governance (IPOG) factors that affect governments’ epidemic and pandemic response has increased immensely since 2020, as a result of the COVID-19 pandemic.
- The IPOG terms are used frequently in this body of literature without definitions; definitions for these terms when they do exist are often constrained by qualifications, inconsistent and incongruent with each other.
- Clearer definitions of these terms as well as delineations of their relationships to each other would help make a rapidly growing body of research on government responses to epidemics and pandemics more discernable and comprehensible.

## Introduction

In response to the ongoing COVID-19 pandemic, government responses to control and manage the disease have varied both in terms of the public health and social measures enacted as well as in the intensity of the implementation of measures (Hale *et al.*, 2022). This has led to a variety of health and social outcomes, where some jurisdictions were more successful in containing the disease while others were less so—despite often implementing the same interventions at what appeared to be similar stages of disease progression. It is clear that contextual elements, such as population demographics, social arrangements, preparedness, infrastructure capacities and citizen cooperation, among others, are contributors to determining the efficacy of epidemic and pandemic response (Berman *et al.*, 2021). ‘Upstream’ or ‘contextual’ factors may determine how the processes producing public health responses work, e.g. in terms of how scientific evidence is used to drive action as well as communication and to persuade the population to comply with recommended interventions (Berman *et al.*, 2021).

Even before the COVID-19 pandemic, it has been argued that, in North America in particular, the focus of public health towards biomedical factors and interventions over the social structures of illness and disease—such as the actions and compositions of governments—has antecedents going as far back as the advent of modern germ theory and bacteriology in the late 19th and early 20th centuries. Public health historians have described this time, and much of the 20th century as well, as an era when public health academics and practitioners alike shifted away from collectively-aimed social reform and towards ‘scientific and technical remedies’ for individuals (Brandt and Gardner, 2000), increasingly in the realm of clinicians and biomedicine rather than in other fields (Fairchild *et al.*, 2010; Jones *et al.*, 2021). A 2018 review calling for more attention ‘taking account of context’ (Craig *et al.*, 2018) is a valuable source on this topic which provides a number of additional, relevant references.

Our research team at the authors’ institute has focused on several of these upstream, contextual determinants that shape public health responses to epidemics and pandemics such as COVID-19, characterized as **i**nstitutes, politics, organizations and governance (IPOG). Our analytical framework focuses on governance (as decision-making processes) at the interface between politics and organization, influenced by wider contextual factors and institutions, which are defined broadly as social norms and rules affecting the behaviour of actors (see Brubacher *et al.*, 2022 for a graphical representation of this framework).

However, previous authors have noted that these factors have been poorly defined, measured and understood, even if they are given mention in passing. For example, previous research on governance in health systems more broadly has noted that ‘the literature on health systems governance is still unfettered at large’ (NTR *et al.*, 2019) and that governance has been ‘an elusive concept to define, assess, and operationalize’, resulting in an overall ‘conceptual chaos’ (Barbazza and Tello, 2014). Indeed, David Levi-Faur in *The Oxford Handbook of Governance* (Levi-Faur, 2012), describing governance more broadly, notes that the ‘notion of governance … was rarely used and nearly incomprehensible before the 1980s, and that ‘the origins, meanings, significance, and implications of the concept of governance are often disputed’. Additionally, literature focused on institutions (Everts, 2013; Rocco *et al.*, 2020; Béland *et al.*, 2021; Huang, 2021), politics (Brahmbhatt and Jonas, 2015; Greer and Singer, 2017; Fowler *et al.*, 2021), organizations (Bardosh *et al.*, 2017), governance (Jönsson and Jönsson, 2012; Honigsbaum, 2017; Huang *et al.*, 2020; Choi *et al.*, 2021), or a combination thereof in relation to epidemic and pandemic response, frequently utilizes these terms, though without clearly defining or operationalizing their use.

The purpose of conducting this research emerged when we found a paucity of COVID-19 and pandemic-related literature in the social, health and biomedical sciences that situates findings in IPOG contexts beyond public health interventions and health outcomes. To this end, the following question has been posed for the conceptual mapping scoping study: In the context of epidemic and pandemic response, how have researchers previously defined IPOG factors? By addressing this question, we aim to enumerate, summarize diverse findings and critically analyse prior definitions of these four terms in the context of epidemic and pandemic response, in order to create more conceptual clarity for these terms and to better inform a common base of knowledge and reference to draw upon in discourse around IPOG. This review also supports our current and future work using these terms in gathering evidence on them and analysing their influence on public health responses in a wide range of jurisdictions.

## Methods

This conceptual mapping scoping study is part of a broader multidisciplinary case study investigating the drivers of health systems’ response to COVID-19 and other health emergencies through a framework developed at the authors’ institute by its interdisciplinary Working Group on Health Systems Response to COVID-19 (Brubacher *et al.*, 2022). Although our research team developed a priori definitions and operationalizations of the terms institutions, politics, organizations and governance, we have sought to clarify our own use of these terms and contribute conceptual clarity to the broader literature.

As such, this research follows guidance from the Joanna Briggs Institute (Peters *et al.*, 2015) as well as the Preferred Reporting Items for Systematic Reviews and Meta-Analyses extension for Scoping Reviews Checklist in supplementary Appendix 1 (see online supplementary material) (Tricco *et al.*, 2018). A scoping review was chosen as an appropriate method to investigate the temporal and geographic scope of articles on IPOG and epidemic and pandemic response. More significantly, conceptual mapping is a more specific type of scoping review methodology (Anderson *et al.*, 2008) that is very fitting for exploring the ways in which prior research has defined and operationalized the concepts of institutions, politics, organizations and governance in our study.

### Search strategy

The following four databases were searched, with the intention of capturing a wide variety of perspectives from medicine and the social sciences as well as grey literature: JSTOR, PAIS, Web of Science and Ovid Medline. This decision to capture a variety of interdisciplinary perspectives reflects our IPOG conceptual framework. Further, our search strategy was designed to capture literature across all geographies, including high-income and low- and middle-income countries. The search strategy was developed in collaboration with a librarian at the authors’ institute, as well as the broader Working Group, all of whom advised on databases to search (Table 1). While some forms of grey literature, such as research reports, were captured through database searches, no additional systematic search of the grey literature was conducted.

**Table 1.** Search string used in Web of Science™ and Ovid Medline® databases and adapted to other databases to retrieve relevant records for this study (see supplementary Appendix 2 in the supplementary material for a comprehensive list of search strings used in each database)

Category	Terms
‘IPOG’ terms	(Institution* OR legal OR enforc* OR organi? ation OR politc* OR ideolog* OR elect* OR policy OR decision OR sociopolitical OR govern* OR regulation* OR regulatory) **AND**
Health crisis terms	(Pandemic* OR ‘infectious disease event’ OR epidemic*) **AND** (‘state of emergenc*’ OR ‘declaration adj4 emergenc*’ OR ‘public health crisis’ OR ‘public health emergenc*’ OR ‘state adj4 emergenc*’) **NOT** (opioid OR opinion OR pre-pandemic OR survey)

Searches were conducted in English with restrictions towards articles and research reports if allowed by the database. There were no restrictions related to study design. Furthermore, the search language was written to limit the focus of the study towards responses to infectious diseases, and exclude responses to non-infectious health crises, such as the opioid crisis, which is often characterized as an epidemic as well (US Department of Health and Human Services, 2021).

The initial scope of the search was without date restrictions and was bounded only by the day upon which the search was conducted in 2021. Due to functional limitations on some of the databases, searches on studies published prior to 2011 were limited to PAIS and Ovid Medline. The impetus for the lack of date restrictions on the search was to include literature about other pandemics and epidemics beyond COVID-19, such as H1N1 and SARS. An updated search was conducted on 8 April 2022, using the same search strings on the same databases, but with an updated date range from 27 July 2021 to 8 April 2022.

Results from each database were exported as .ris files and imported into *Covidence* systematic review software. Additionally, upon recommendation from senior members of the Working Group on Health Systems Response to COVID-19 at the authors’ institute, the following topically-relevant journals were hand-searched:

- *Public Administration Review*
- *Journal of Health Politics, Policy and Law*
- *Journal of Comparative Policy Analysis: Research and Practice*
- *Nature*
- *BMJ Global Health*

with one article each from *Public Administration Review* and *Journal of Comparative Policy Analysis: Research and Practice* ultimately being included in the final results.

### Eligibility and screening

Two reviewers independently screened the retrieved records for eligibility using *Covidence.* For Level 1 (title/abstract) screening, eligibility was guided by the following questions: (1) Does the article discuss at least one of institutions (I), politics (P), organizations (O) or governance (G)^1^ in the context of public health crisis management or preparedness? (2) Is the article in English? Those articles meeting both criteria proceeded to Level 2 (full-text) screening. Sample research topics included/excluded based on these criteria are noted in Supplementary Appendix 3, see online supplementary material.

In Level 1 screening, we identified that our search strategy was highly sensitive—and therefore retrieved many articles—but not necessarily specific enough. To manage the scope of the research, and ensure we were meeting our research objectives, we further refined our eligibility criteria after Level 1 to be more stringent in our exclusion of articles not pertinent to our objectives. As such, Level 2 screening was guided by the following criteria: Does the article define AND use^2^ at least one of the IPOG terms in relation to public health crisis management or preparedness? Reviewers discussed all conflicts until consensus regarding inclusion or exclusion was reached.

### Data extraction and analysis

Two independent reviewers extracted the following data from each included article: authors/year/title; study location; disciplinary lens(es); methodology (quantitative/qualitative/mixed), IPOG factor(s) defined and used; definition/operationalization of IPOG term(s) in the study; and the reported impact of the IPOG factor(s) on public health crisis management and/or preparedness (supplementary Appendix 4, see online supplementary material). Conflicts between reviewers were again resolved by discussion and consensus as to the extracted content.

Basic descriptive statistics (e.g. counts and proportions) were calculated across articles. Extracted data were analysed thematically, using a constant comparative method (Braun and Clarke, 2006). *QSR NVivo®* software was used for organization of codes and retrieval of coded excerpts from articles. A hybrid of inductive and deductive coding was used to identify and categorize excerpts from articles according to their use of IPOG terms, as well as subcategories of themes within each of those four terms. Inductive coding was used to separate excerpts in relation to observations by the reviewers, while deductive coding was used in mapping some excerpts onto previously noted definitions, in particular conceptions of governance used in the *Oxford Handbook of Governance.*

## Results

### Temporal and geographic scope of articles

**Figure 1.**
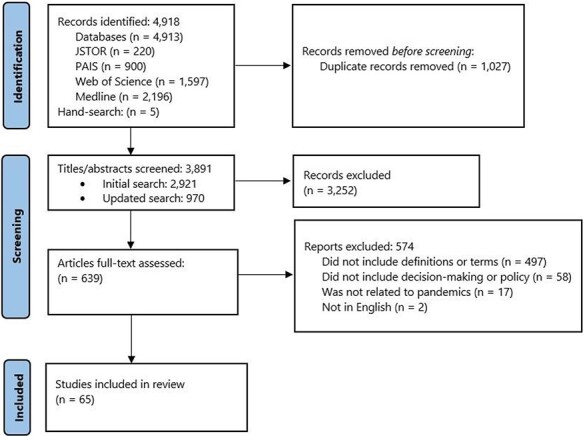
Diagram depicting the flow of identification, screening and eligibility assessment of articles included in this study

In total, 65 publications met the eligibility criteria and were included in the review (Figure 1): 16 involved discussion of institutions, 17 involved politics, 6 involved organizations and 34 involved governance. Eight of the included publications (12.3%) discussed multiple terms^3^. A list of included articles can also be seen in in Table 2.

**Table 2.** List of included articles retrieved from a systematic search of the published academic literature on IPOG factors in epidemic and pandemic response. Articles are organized chronologically by year of publication, with author(s) title, study region(s), and factor(s) studied also indicated

Author(s) and year	Title	Region(s)^a^	Term(s) defined
Wilson and Lazar, 2006	From SARS to Avian flu—why Ottawa must lead Canada’s response	Americas	Governance
Nguyen, 2009	Government-by-exception: Enrolment and experimentality in mass HIV treatment programmes in Africa	Africa; multilateral	Governance
Hoffman, 2010	The evolution, etiology and eventualities of the global health security regime	Multilateral	Governance
Gerwin, 2011	Planning for pandemic: a new model for governing public health emergencies.	Americas	Governance
Kamradt-Scott, 2013	The politics of medicine and the global governance of pandemic influenza	Multilateral	Governance
Hanrieder and Kreuder-Sonnen, 2014	WHO decides on the exception? Securitization and emergency governance in global health	Multilateral	Governance
Carney and Bennett, 2014	Framing pandemic management: New governance, science or culture?	Oceania; multilateral	Governance
Roodenrijs *et al.*, 2014 * *	Risk governance for infectious diseases: exploring the feasibility and added value of the IRGC-framework for Dutch infectious disease control	Europe	Governance
Calain and Abu Sa’Da, 2015	Coincident polio and Ebola crises expose similar fault lines in the current global health regime	Africa; multilateral	Governance
Hirsch *et al.*, 2015	Caught in the Middle: The Contested Politics of HIV/AIDS and Health Policy in Vietnam	Asia	Governance
Mack *et al.*, 2016	Global Health Risk Framework: Governance for Global Health Workshop Summary	Multilateral	Governance
Gostin and Katz, 2016	The International Health Regulations: The Governing Framework for Global Health Security	Multilateral	Governance
Speakman *et al.*, 2017	Pandemic legislation in the European Union: Fit for purpose? The need for a systematic comparison of national laws	Europe; multilateral	Governance
Wenham, 2018	Regionalizing Health Security	Asia; multilateral	Governance
Zhang and Zhang, 2020	COVID-19 in China: Power, Transparency and Governance in Public Health Crisis	Asia	Governance
Kim *et al*., 2020a * *	Assessing the South Korean Model of Emergency Management during the COVID-19 Pandemic	Asia	Governance
Christensen and Lægreid, 2020	Balancing governance capacity and legitimacy—how the Norwegian government handled the COVID-19 crisis as a high performer	Europe	Governance
Collins *et al*., 2021 * *	Addressing the double burden of the COVID-19 and noncommunicable disease pandemics: a new global governance challenge	Multilateral	Governance
Agartan *et al*., 2020	Introduction: COVID-19 and WHO: Global institutions in the context of shifting multilateral and regional dynamics	Multilateral	Governance
Coco and Dias, 2020	Prevent, Respond, Cooperate States’ Due Diligence Duties vis-a-vis the covid-19 Pandemic	Multilateral	Governance
Zhou *et al.*, 2020 * *	Global health governance for travel health: lessons learned from the coronavirus disease 2019 (COVID-19) outbreaks in large cruise ships	Multilateral	Governance
Finnane, 2021	Governing in a Pandemic: Law and Government in Australia, 1919	Oceania	Governance
Areal and Sheppy, 2021	A Crisis of Governance—Or an Opportunity?	Europe	Governance
Thomson and Ip, 2020	COVID-19 emergency measures and the impending authoritarian pandemic	Africa; Americas; Asia; Europe; Oceania; multilateral	Governance
Meszaros, 2021	Carl Schmitt in Hungary: Constitutional Crisis in the Shadow of Covid-19	Europe	Governance
Shultz and Viczko, 2021	What are we saving? Tracing governing knowledge and truth discourse in global COVID-19 policy responses	Multilateral	Governance
Wang *et al.*, 2021 * *	Community Resilience Governance on Public Health Crisis in China	Asia	Governance
Zhang and Wang, 2021	Administrative Governance and Frontline Officers in the Chinese Prison System During the COVID-19 Pandemic	Asia	Governance
Yao *et al.*, 2021	Interactive Governance Between and Within Governmental Levels and Functions: A Social Network Analysis of China’s Case Against COVID-19	Asia	Governance
Windholz, 2020	Governing in a pandemic: from parliamentary sovereignty to autocratic technocracy	Oceania	Institutions; governance
He *et al*., 2020	Crisis governance, Chinese style: distinctive features of China’s response to the Covid-19 pandemic	Asia	Institutions; governance
Goetz and Martinsen, 2021	COVID-19: a dual challenge to European liberal democracy	Europe	Institutions; governance
Doyle, 2006	An international public health crisis: can global institutions respond effectively to HIV/AIDS?	Multilateral	Institutions
McCormick and Whitney, 2013	The making of public health emergencies: West Nile virus in New York City	Americas	Institutions
Rosella *et al*., 2013 * *	Pandemic H1N1 in Canada and the use of evidence in developing public health policies—A policy analysis	Americas	Institutions
Kim, 2015	World Health Organization and Early Global Response to HIV/AIDS: Emergence and Development of International Norms	Multilateral	Institutions
Nohrstedt and Baekkeskov, 2018	Political drivers of epidemic response: foreign healthcare workers and the 2014 Ebola outbreak	Africa	Institutions
De Angelis and de Oliveira, 2021	COVID-19 and the ‘state of exception’: assessing institutional resilience in consolidated democracies—a comparative analysis of Italy and Portugal	Europe	Institutions
West-Oram, 2021	Solidarity is for other people: identifying derelictions of solidarity in responses to COVID-19	Europe	Institutions
Yan *et al*., 2021 * *	Culture, Institution, and COVID-19 First-Response Policy: A Qualitative Comparative Analysis of Thirty-One Countries	Asia; Europe; Oceania; multilateral	Institutions
Soon *et al*., 2021	Withstanding the plague: Institutional resilience of the East Asian welfare state	Asia	Institutions
Fei, 2022	Assembling Chinese health engagement in Africa: structures, strategies and emerging patterns	Africa; Asia	Institutions
Adeel *et al*., 2020 * *	COVID-19 Policy Response and the Rise of the Sub-National Governments	Americas	Institutions; Politics
Vankovska, 2020	Dealing with COVID-19 in the European periphery: between securitization and gaslighting	Europe	Institutions; Politics
Teigen, 2007	Legislating fear and the public health in gilded age Massachusetts	Americas	Politics
Lawson and Xu, 2007	SARS in Canada and China: Two Approaches to Emergency Health Policy	Americas; Asia	Politics
Burkle, 2020	Declining Public Health Protections within Autocratic Regimes: Impact on Global Public Health Security, Infectious Disease Outbreaks, Epidemics, and Pandemics	Africa; Americas; Asia; Europe	Politics
Ragozina, 2020	Islamic Biopolitics during Pandemics in Russia: Intertextuality of Religious, Medical and Political Discourses	Europe	Politics
Dostal, 2020	Governing Under Pressure: German Policy Making During the Coronavirus Crisis	Europe	Politics
Lee, 2020	WHO under fire: The need to elevate the quality of politics in global health	Multilateral	Politics
Afsahi *et al*., 2020 * *	Democracy in a Global Emergency Five Lessons from the COVID-19 Pandemic	Multilateral	Politics
Moulds, 2020	Scrutinising COVID-19 laws: An early glimpse into the scrutiny work of federal parliamentary committees	Oceania	Politics
Sandset, 2021	The necropolitics of COVID-19: Race, class and slow death in an ongoing pandemic	Europe	Politics
Nelson, 2021	Pandemic Politics in South Asia: Muslims and Democracy	Asia	Politics
Biehl *et al*., 2021	Supreme Court v. Necropolitics: The Chaotic Judicialization of COVID-19 in Brazil	Americas	Politics
Fowler *et al*., 2021	Pandemics and Partisanship: Following Old Paths into Uncharted Territory	Americas	Politics
Hier, 2021	Narrating the crisis: Moral regulation, overlapping responsibilities and COVID-19 in Canada	Americas	Politics
Liu *et al*., 2020	Striking a balance between science and politics: understanding the risk-based policy-making process during the outbreak of COVID-19 epidemic in China	Asia	Politics; governance
Makarychev and Romashko, 2021	Precarious Sovereignty in a Post-liberal Europe: The COVID-19 Emergency in Estonia and Finland	Europe	Politics; governance
Liu, 2004	China’s public health-care system: facing the challenges	Asia	Organizations
Schwartz *et al*., 2007	Evolution of Health Provision in Pre-SARS China: The Changing Nature of Disease Prevention	Asia	Organizations
Forestier *et al*., 2016	Coordination and relationships between organisations during the civil-military international response against Ebola in Sierra Leone: an observational discussion	Asia	Organizations
Boyce and Katz, 2021	COVID-19 and the proliferation of urban networks for health security	Multilateral	Organizations
Kim *et al*., 2020b	From Uncoordinated Patchworks to a Coordinated System: MERS-CoV to COVID-19 in Korea	Asia	Organizations
Liu *et al*., 2021 * *	Multi-Level Governance, Policy Coordination and Subnational Responses to COVID-19: Comparing China and the US	Americas; Asia	Institutions; organizations

a Regions are based upon the UN Geoscheme.  IRGC, International Risk Governance Council added; ‘multilateral’ refers to articles that studied entities with no geographic borders, such as the UN, WHO or World Bank.

**Figure 2.**
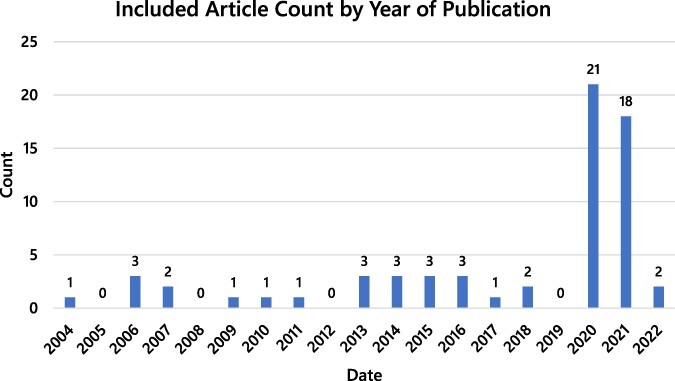
Summary of the number of publications per year (2004–2022) related to IPOG factors affecting epidemic and pandemic response

**Figure 3.**
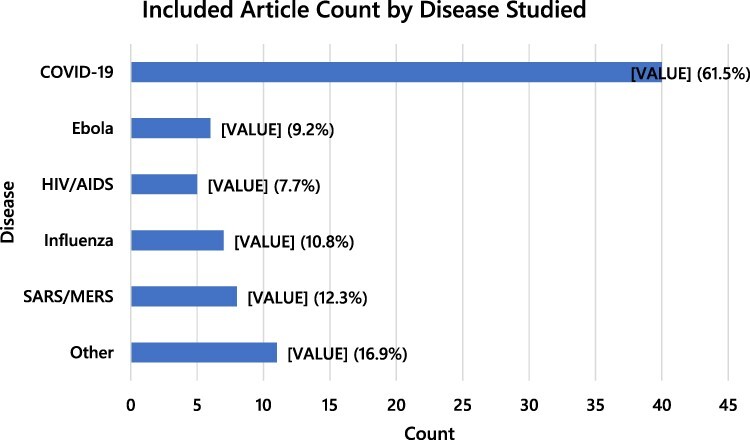
Article counts by disease studied. Note that totals exceed 65 and proportions exceed 100%, as some articles discussed multiple diseases

Included articles were published between 2004 and 2022. The majority of articles were published in 2020 or 2021 (60%) or focused on the COVID-19 pandemic (61.5%). No differences were observed in how IPOG terms were conceptualized in relation to pandemics vs epidemics. A visualization of the chronology of included articles can be seen in Figure 2 and the diseases of focus in Figure 3, keeping in mind that some articles discussed multiple diseases.

Please correct [VALUE] errors. The correct values are: COVID-19 (40), Ebola (6), HIV/AIDS (5), Influenza (7), SARS/MERS (8), Other (11). The figures have been re-uploaded into iAuthor on a Word document for reference.

In addition, there was a wide geographic scope pertaining to the jurisdictions studied in the included literature. A large proportion of articles studied jurisdictions in Asia, Europe or international groups such as the United Nations (32.3, 24.6 and 33.8%, respectively), while jurisdictions in Africa were studied the least (9.2%). The distribution of article count by geographic region is seen in Table 3, keeping in mind that some articles discussed multiple geographic areas.

**Table 3.** Counts and proportions of articles by region of focus. Regions are based upon the UN Geoscheme

Region name	Article count	Article proportion^a^
Africa	6	9.2%
Americas	13	20%
Asia	21	32.3%
Europe	16	24.6%
Oceania	6	9.2%
Multilateral^b^	22	33.8%

a Proportions and counts exceed 100% as some articles examined jurisdictions in multiple regions.

b Articles that studied entities with no geographic borders, such as the United Nations, WHO or World Bank.

### How were institutions, politics, organizations and governance defined and operationalized in the literature?

Articles defined and operationalized the concepts of IPOG in varied ways. Individual results for each term follow below. For each term, examples of definitions and operational usage from the literature are provided, along with descriptions and reasoning behind the sub-categorizations they have been placed in within a wider term.

### Institutions

The first three listed definitions are one of two broad categories in which the term ‘institutions’ was used in the context of epidemic and pandemic responses in the retrieved literature—a sense of ‘institutions’ as norms, rules, ideas and processes within a system. The fourth definition is an example of the second category, i.e. the use of the term ‘institutions’ as a synonym for organizations, as well as the structures and systems within which those organizations exist. Examples of how ‘institutions’ were defined in the included literature can be viewed on Table 4.

**Table 4.** Examples of definitions for ‘institutions’ in the included literature

Examples of definitions for ‘institutions’	1. ‘Institutional rearrangement, draws attention to the processes through which the policies and procedures put in place during emergency declarations ramify, and permanently change the face of public health governance.’ (McCormick and Whitney, 2013)
	2. ‘Factors that create such continuity and predictability are commonly labelled institutions and include rules, norms, widely shared ideas, and other enduring socioeconomic and political structures that mould behaviour’ (Nohrstedt and Baekkeskov, 2018)
	3. ‘Solidarity is fully institutionalised, ‘in the form of legally enforceable norms’, such as progressive tax systems and welfare state arrangements’ (West-Oram, 2021)
	4. ‘Institutional context refers to the systems and processes that countries use to structure authority, attention, information flows, and relationships in addressing policy problems … institutional factors are concerned with the formal power structure, legal system, and regulations, whereas culture orientation emphasizes the informal norms, beliefs, values, and customs’ (Yan *et al*., 2021)

In the first sense, norms, rules, ideas, structures and/or processes were made present through mechanisms of ‘institutionalization’, either informally through guiding actors’ behaviours (Rosella *et al.*, 2013; Kim, 2015; Nohrstedt and Baekkeskov, 2018; De Angelis and de Oliveira, 2021) or formally through structures such as taxation or constitutions (Rosella *et al.*, 2013; Windholz, 2020; West-Oram, 2021; De Angelis and de Oliveira). Similarly, the concept of institutions as organizations could take place formally or informally—in the sense of the former, discrete organizations such as the Public Health Agency of Canada (PHAC), the Global Fund, the World Health Organization (WHO), state-owned enterprises or the World Bank (Doyle, 2006; Adeel *et al.*, 2020; Fei, 2022); in the sense of the latter, as ‘structure[s of] authority, attention, information flows, and relationships’, informal counterparts to ‘formal executive, legislative, and bureaucratic structures of public health’ (Rosella *et al.*, 2013; Yan *et al.*, 2021). The intermixing of the terms ‘institutions’ and ‘organizations’ to refer to the same concept especially highlights the fluidity with which these terms have been defined by various authors. For example, the prior practice of organizational mapping has taken place with reference only to ‘institutional structure[s]’, rather than ‘organizations’ (Schwartz *et al.*, 2007; Liu *et al.*, 2021).

### Politics

In the examined articles, a strict definition of politics, as well as a conception of what ‘good politics’ might be, was found in only one article (Lee, 2020), even though 17 articles ultimately included in the extraction discussed politics. In this article, ‘politics’ on its own, without any prefixes or qualifiers, was defined as ‘the relationships within a group or organization that allow particular people to have power over others’ and ‘how decisions are made within such groups or organizations including how shared goals are agreed and achieved’. From there, the notion of ‘good politics in global health’ is described as ‘adherence to principles of good governance; that is, the extent to which rules and procedures are built on principles of transparency and accountability, effectiveness, representativeness and participation, and rule of law’.

The last characterization especially aligns with the more commonplace conceptions or characterizations of politics in relation to epidemic and pandemic response seen in the captured literature that focused on politics. Such articles fall under two broad categories: descriptions of what structures comprise the domain of politics and discussions of how they function in practice—legislative committees, political parties, drafting of legislation, partisan behaviour—and value-based assessments of these structures and practices, such as ‘good politics’ and the relationship between power and agency over one’s death.

Returning to this conception of what ‘good politics’ might be, as mentioned previously (Lee, 2020), it is clearly stated in the same article that the ‘spaces’ in which politics take place are ‘making (legislature), implementing (executive), and enforcing (judiciary)’. Other articles similarly describe places which the authors conceive of as spaces for politics to take place—legislative action (Teigen, 2007; Adeel *et al.*, 2020; Afsahi *et al.*, 2020; Nelson, 2021), participation in a political system (Burkle, 2020), the composition of a legislative committee (Moulds, 2020), as well as partisanship and coalition-forming between political parties (Fowler *et al.*, 2021; Nelson, 2021).

In the context of epidemic and pandemic response, the concept of politics as values in part took the approach of describing the regulation of life and mortality as a political matter (Teigen, 2007; Ragozina, 2020; Biehl *et al.*, 2021). Of note here is the continued association of politics with power in this characterization, such as defining ‘necropolitical’ as the subjugation of ‘life to the power of death’ (Biehl *et al.*, 2021) and the way in which ‘particular groups and communities are relegated to zones of living that are not life-giving but conditions of slow death… either through poverty, detrimental working conditions, nutrition and pollution (Sandset, 2021), as well as the Foucauldian notion of biopolitics, and by extension, biopower. Biopolitics in the captured literature is defined in the sense of states governing mortality in times of disease—through gaining authority to assign subjects into statuses of infected or uninfected (Lawson and Xu, 2007), the ‘moralization of health’ through measures aimed at individuals, such as vaccine uptake (Hier, 2021), as well as ‘the political regulation of corporality’ and individuals’ bodies through disease control measures (Ragozina, 2020).

### Organizations

Out of the four terms examined in this conceptual mapping scoping study, ‘organizations’ had the smallest number of articles captured in the search, a total of 6. In most of these articles, analysis of the structure and functions of organizations involved within a public health response, such as non-governmental organizations, government agencies or components within agencies, involved a form of organizational mapping of health systems (Liu, 2004; Schwartz *et al.*, 2007), emergency response structures (Forestier *et al.*, 2016; Kim *et al.*, 2020b; Liu *et al.*, 2021) and the ways in which these structures have changed over time (Schwartz *et al.*, 2007; Kim *et al.*, 2020b). This was akin to one of the definitions of governance proposed by Levi-Faur, that of governance as structure: e.g. ‘horizontal’ or ‘vertical’ arrangements (Barbazza and Tello, 2014); ‘vertical integration’ of health system components is also described as a type of ‘organizational strategy’ for reform (Liu, 2004).

Relative to other terms, especially ‘governance’ and ‘institutions’, strict definitions for the term ‘organizations’ itself were sparse in the included literature. More frequent were descriptions of specific examples of organizations: an epidemic prevention station, county health bureaus (Schwartz *et al.*, 2007), aid groups such as the United Nations Development Programme (Forestier *et al.*, 2016), or even formal groups such as the Global Parliament of Mayors with ‘established patterns of communication, policy-making and exchange’ (Boyce and Katz, 2021). One included article was able to meet the previously listed conditions—organizational mapping of changes to a health system over time, describing the role and functions of each component and their relationship to each other, and placing the system in the context of its response to an infectious disease outbreak—while using the term ‘organization’ exclusively in reference to the WHO (Schwartz *et al.*, 2007).

### Governance

The use of the term ‘governance’ in the included literature was both the most numerous of the four terms—with 34 articles (52.3%) providing a definition and operationalization of the term—and also the most varied in the ways in which researchers have previously defined these terms. We decided to categorize definitions of governance used by their scale of complexity, ranging from perceptions of governance as the structure of organization(s) involved in the response to a public health emergency, distinct consideration of processes within those structures as part of one’s conception of governance, to perceiving governance as changes in structure and processes as part of mechanisms to improve responses, which could include broader reforms to structure and processes as an overall strategy guiding a jurisdiction’s epidemic and pandemic response. Examples of how ‘governance’ was defined in the included literature can be viewed on Table 5.

**Table 5.** Examples of definitions for ‘governance’ in the included literature

Examples of definitions for ‘governance’	1. ‘In the context of infectious disease outbreaks of global significance, governance encompasses a range of integrated policy, information management, command, and control mechanisms for facilitating collective action to achieve the objectives of prevention, detection, and response. Of necessity, these mechanisms integrate actions across intergovernmental organizations, sovereign nations, communities, the corporate sector, humanitarian agencies, and civil society. They operate in not only the realm of health, but also to a variable extent in collateral spheres to include agriculture/food security, diplomacy, education, finance, migration/refugee care, security, and transportation.’ (Mack *et al.*, 2016)
	2. ‘The way in which the global health systems are managed’ (Mack *et al.*, 2016)
	3. ‘The organized social response to health conditions at the global level.’ (Mack *et al.*, 2016)
	4. Governance capacity as ‘[The] preparedness or analytical capacity, coordination, regulation and implementation or delivery capacity … to provide effective crisis management’, and governance legitimacy as ‘citizens’ trust in government and concerns such issues as accountability, support, expectations, and reputation’ (Christensen and Lægreid, 2020)
	5. ‘Governance refers to the steering of society with regard to societal problems. Risk governance can be defined as ‘both the institutional structure and the policy process that guide and restrain collective activities of a group, society or international community to regulate, reduce or control risk problems’’ (Roodenrijs *et al.*, 2014)
	6. Global health governance is defined as ‘the use of formal and informal institutions, rules, and processes by states, intergovernmental organizations, and nonstate actors to deal with challenges to health that require cross-border collective action to address effectively’ (Zhou *et al*., 2020; Collins *et al*., 2021)
	7. Adaptive governance is defined as flexible and learning-based multi-level modes of governance or institutional arrangements that can build resilience for the challenges posed by complex and urgent problems (Kim *et al*., 2020a)
	8. ‘Global social governance [is] the mechanisms that enable the international community to address global social problems, through systems of global regulation across national borders and a globally agreed set of social rights’ (Agartan *et al*., 2020)
	9. ‘The assignment of authority and the specification of procedures’ (Speakman *et al*., 2017)
	10. ‘[Multi-level governance] is defined as a governance system within which power is dispersed across government levels vertically and across sectors horizontally’ (Yao *et al.*, 2021)
	11. ‘Multi-level governance refers to the institutional arrangements of policy making and implementation that involve continuous interaction and coordination among government and non-government actors across different levels and territories … Type I referring to a system of power sharing among different levels of general-purpose jurisdictions and Type II being essentially a polycentric system of decentralized, overlapping, and competitive jurisdictions’ (Liu *et al*., 2021)
	12. ‘Corporate governance ‘is concerned with the structures and systems of control by which managers are held accountable to those that have a legitimate stake in an organisation’’ (Areal and Sheppy, 2021)

References to structures of governance alone in the captured literature are comparatively limited, and are largely focused on higher-level complex organizations such as the WHO (Carney and Bennett, 2014), frameworks such as the International Health Regulations (IHR) (Collins *et al.*, 2021) or the structures of government hierarchies within national jurisdictions (Liu *et al.*, 2020; Wang *et al.*, 2021; Finnane, 2021). A description of a hypothetical governance structure to be used in a public health emergency can be seen in a proposed structure for governance during public health emergencies, in the form of an ‘emergency republic’ comprised of an independent technocratic agency of advisory experts akin to the American Federal Reserve, the relegation of the legislature to monitoring government accountability for the executive and the passage of emergency powers from the executive to the independent technocracy (Gerwin, 2011). The similarities between this conception of governance as structures alone means that many other articles that discuss similar content go by other names, such as ‘organizations’ and ‘institutions’, also discussed in this research.

In the included literature which conceptualized governance as processes within those structures, organizations such as the World Influenza Center (Kamradt-Scott, 2013) or the Association of Southeast Asian Nations (Wenham, 2018) as well as agreements such as the IHR (Wilson and Lazar, 2006; Gostin and Katz, 2016; Zhou *et al.*, 2020; Collins *et al.*, 2021) were also discussed. However, in addition to descriptions of the existence of such structures or their base setups, articles in this category provided further context as to the interactions of various processes with one another, such as the ways in which subnational surveillance, national assessments and WHO assessments interact with one another in the WHO director-general’s decision to declare a Public Health Emergency of International Concern, visualized on a flow chart (Gostin and Katz, 2016). An article on pandemic legislation in the European Union defined governance as ‘the assignment of authority and the specification of procedures’ (Speakman *et al.*, 2017).

Many articles also sought to describe processes of governance within the jurisdiction of a single state in the context of a single disease at both national and subnational levels of government. Processes characterized as ‘governance’ by their authors include matters such as collaboration and coordination between local, state/provincial and federal/central levels of government in the USA and China respectively during the COVID-19 pandemic (Zhang and Zhang, 2020; Liu *et al.*, 2021), the ‘preparedness or analytical capacity, coordination, regulation and implementation or delivery capacity’ of Norway’s government in response to COVID-19 (Christensen and Lægreid, 2020), the use of enabling acts in transferring legislative power to an executive in times of emergency (Meszaros, 2021), as well as the use of technology as a method by which to practice the act of governing and exercising authority, particularly through the use of online technologies to achieve societal functions remotely during responses to a disease (Nguyen, 2009; Shultz and Viczko, 2021).

Governance in the included literature was also seen as including procedures of decision-making, such as ability to issue commands in a hierarchy. This is seen particularly in multilateral organizations such as the WHO (Carney and Bennett, 2014; Agartan, Cook and Lin 2020) and in the particular context of strong top-down centralized governance mechanisms in the People’s Republic of China (He *et al.*, 2020; Zhang and Zhang, 2020; Zhang and Wang, 2021). The included literature describes changes in processes for decision-making in epidemic and pandemic response over time: e.g. example moving away from exchanges and deliberation between jurisdictions towards ‘member states ceding “a considerable part of their respective sovereignty in national public health policy to the international community”’ to increasingly structured and litigated groups such as the WHO (Wilson and Lazar, 2006). One article characterized the WHO’s role in global health governance as ‘hegemonic’ due to near-universal participation in it by the countries of the world, its displacement of states as the exclusive authority in certain international affairs, and the ways in which WHO-related instruments such as the IHR instruct member states to act with regard to infectious disease control, albeit without enforcement mechanisms (Hoffman, 2010).

Values-based assessments of governance structures, processes and mechanisms are used in some literature, such as descriptors like ‘good governance’. In the context of epidemic and pandemic response, these frequently relate to exceptional conditions of governance during times of pandemic, going beyond ‘normal’ governing processes, and include an implied positive valuation with outcomes in the case of adoption of approaches for governing for the purposes of bolstering a jurisdiction’s sense of security (Hanrieder and Kreuder-Sonnen, 2014; Calain and Abu Sa’Da, 2015; Wenham, 2018).

The ways in which the local historical context of jurisdictions inform responses to health crises is mentioned as well and given an alternative values-based characterization as something other than ‘good governance’. For example, both Vietnam and China are noted to employ a ‘Leninist mode of governance’ in their response to HIV/AIDS and COVID-19 respectively (Hirsch *et al.*, 2015; He *et al.*, 2020); Chinese response to COVID-19 is especially noted in its use of militaristic rhetoric and fast-tracked promotions for well-performing local Communist Party (CPC) cadres involved in response. Both practices date back to the foundation of the People’s Republic on the basis of armed revolution, and in particular, the latter practice of fast-tracked promotions for civilian cadres has a basis in ‘battlefield promotions’ for military personnel when the CPC was not yet in government in China prior to 1949 (He *et al.*, 2020).

A modality of governing particularly worth mentioning because of its direct relation to other works that provide discrete definitions for terms is the Foucauldian notion of ‘governmentality’ (Foucault, 1991). In the context of epidemic and pandemic response, this was often used in the included articles in relation to the application of technology and the ‘managerial and administrative capacities’ of governments towards managing human life, as well as the discrete functions of states during a pandemic, such as online learning technologies and the administration of social welfare (Makarychev and Romashko, 2021; Shultz and Viczko, 2021). In particular, his concept of ‘governing knowledges’ discusses a philosophy of governing based primarily on practice, action and technology, rather than theory or ideology (Shultz and Viczko, 2021). In all, five of the included articles used the framing of ‘governmentality’ in the context of governance during pandemics and epidemics (Lawson and Xu, 2007; Nguyen, 2009; Vankovska, 2020: Makarychev and Romashko, 2021; Shultz and Viczko, 2021).

Also falling under the purview of governance strategies would be the practice of using values such as ‘democratic’, ‘smart’ or ‘good’ in defining ideal practices of governance, as noted in previous reviews on health governance more generally (Barbazza and Tello, 2014). Articles in this review utilized concepts such as ‘adaptive governance’ (Kim *et al.*, 2020a), ‘global social governance’ (Agartan, Cook and Lin 2020), ‘authoritarian governance’ (Thomson and Ip, 2020), ‘community public health crisis governance’ (Wang *et al.*, 2021), ‘multi-level governance’ (Liu *et al.*, 2021; Yao *et al.*, 2021) and the aforementioned ‘good governance’ (Coco and Dias, 2020; Lee, 2020; Kim *et al.*, 2020a). Definitions of these values ranged from the descriptive, such as ‘adaptive governance’ being defined as ‘flexible and learning-based multi-level modes of governance or institutional arrangements that can build resilience for the challenges posed by complex and urgent problems’, to the tautological, where the ‘principles of good governance’ were used as measurements against the notion of ‘good politics’ (Lee, 2020).

Finally, it is worth noting here a tendency for reviewed articles to preface definitions with a prefix or qualifier, either to convey values (such as ‘good’ governance) or describe a particular typology, such as a horizontal or vertical structure of governance (Barbazza and Tello, 2014). Although this took place most frequently with governance—where the value-based conception of ‘good governance’ has appeared frequently (Coco and Dias, 2020; Lee, 2020; Kim *et al.*, 2020a) along with descriptors such as ‘emergency’, ‘adaptive’, ‘administrative’ governance, and so on (Agartan, Cook and Lin 2020; Wilson and Lazar, 2006; Hanrieder and Kreuder-Sonnen, 2014; Thomson and Ip, 2020; Kim *et al.*, 2020a; Liu *et al.*, 2021; Wang *et al.*, 2021; Zhang and Wang, 2021)—this tendency has also appeared with regards to the terms ‘institutions’ and ‘politics’.

## Discussion

### Rapid and recent growth in the literature and bias towards higher-income countries

Although the search had no limitation on the lower end of the date range, we still found that a majority of the reviewed literature on IPOG in epidemic and pandemic response was related to COVID-19 (61.5%), or published in 2020 or 2021 (60%). In prior years, smaller upticks in publication of relevant literature were witnessed congruent with the global spread of H5N1 influenza in 2006/2007 and the 2013–2016 Ebola epidemic in Western Africa, but the cascade of literature related to COVID-19 exceeds both by a very wide margin. Indeed, by late summer of 2021, published literature on COVID-19 has exceeded that of H1N1, Zika, Ebola, HIV/AIDS and even tuberculosis (Ioannidis *et al.*, 2021).

Additionally, it was also noticed that the majority of the reviewed literature had a focus on multilateral groups such as the WHO (33.8%) or higher-income jurisdictions in Europe (24.6%) and Asia (32.3%), while articles that had a focus on jurisdictions in Africa in particular were comparatively scant (9.2%), especially given the disproportionate burden of disease still faced in many parts of the world (GBD 2019 Diseases and Injuries Collaborators, 2020). To some extent this may be another side-effect of the enthusiastic academic response to COVID-19; a heavy focus on European countries in particular has been noted in previous writing on the impact of politics on jurisdictions’ response to COVID-19 (Wu, 2022). Furthermore, the origin of COVID-19 in East Asia, along with its early spread to Europe before other parts of the world (Sanyaolu *et al.*, 2021), might explain the greater degree of attention given to these two regions of the world in the literature at the time of search.

### Institutions

The dual definitions employed for the same term, ‘institutions’—on one hand a synonym for organizations, and on the other as a term to capture a sense of norms, rules, ideas and processes within a system—serve as a suitable initial indication of the fluidity with which these four terms—‘institutions’, ‘politics’, ‘organizations’ and ‘governance’—were found to have been used in relation to each other in the context of epidemic and pandemic responses. In this particular case, what was characterized by some authors as a function of ‘institutions’ could just as easily describe functions of organizations or governance if using the terminology of a different author, or vice versa.

The use of the term ‘institutionalization’ may be seen as exemplifying this blurring of conceptual aspects of ‘institutions’, such as norms, with the ‘structures’ societies put in place to enable or support the realization of institutions. These ‘structures’ are often ‘organizations’ in actuality. For example, respect for the rule of law may be seen as an institution, while the courts are organizations that enable the rule of law to be applied, but they are not themselves the institutions per se. This distinction may be useful when research identifies conflicts or gaps between an ‘institution’ and the ‘organizations’ that exist to support it—e.g. in a situation where courts are corrupt or perceived as not respecting the rule of law. Clarifying such distinctions in both defining these terms and framing them in relationship to each other could be useful in reducing confusion and providing clearer lines of discussion between publications.

### Discussion on politics

Compared to other terms, and especially relative to the degree of discussion on the topic, precise definitions of ‘politics’ were difficult to come by. Indeed, some authors seemed to perceive ‘politics’ as comparatively fluid or subjective by contrasting it with practices perceived as more immutable or steadfast by comparison, e.g. law, science or decisions made by agencies comprised of professional civil servants such as the PHAC (Adeel *et al.*, 2020; Dostal, 2020; Liu *et al.*, 2020), thus evading establishing a firm definition for ‘politics’ itself. In his apprehension in defining obscenity, US Supreme Court Justice Potter Stewart famously wrote ‘I know it when I see it’ (Potter, 1964). In many ways the authors of the captured studies have attempted to use such intuitiveness to define ‘politics’ as well, perhaps attempting to bank upon the ubiquity of the term in daily conversation. However, in practice, the summation of such elusiveness was a sense of reluctance in the literature to define the term outright.

This elusiveness in defining ‘politics’ could also be seen in the way in which politics was characterized as a sense of values in the captured literature; e.g. through characterizing the extent to which a conception of politics adhered to certain values, norms or processes, and by extension act as ‘good politics’. Examples of some of these include ‘principles of transparency and accountability, effectiveness, representativeness and participation, and rule of law’ (Lee, 2020), ‘legitimacy, transparency, accountability, equity, justice, and effectiveness’ (Forum on Medical and Public Health Preparedness for Catastrophic Events *et al.*, 2016), as well as ‘social justice and equity’ (Liu *et al.*, 2020). In some articles, while politics was not defined unto itself, ‘politics’ was contrasted against practices such as science (Liu *et al.*, 2020), ‘the codified legal order’ (Dostal, 2020) or ‘good governance’ (Lee, 2020). There is a sense here that some authors have viewed politics as lacking an inherent value on its own, or even morally suspect, whereas others might see it as a way by which societies structure the processes around the use of power. Discussion of ‘politics’ was not the only situation in which descriptors of value were used; such tendencies were also seen frequently with regards to governance.

Another way in which writers sought to qualify their use of ‘politics’ in relation to epidemic and pandemic response was through the use of prefixes and qualifying terms, either attached to ‘politics’—such as ‘good politics’ or ‘emergency politics’ (Afsahi *et al.*, 2020; Lee, 2020; Ragozina, 2020)—or through the use of ‘politics’ as a qualifying term itself, like ‘political systems’ or ‘political participation’ (Burkle, 2020). An especially common prefix term used in relation to ‘politics’ was the Foucauldian term ‘biopolitics’ (Lawson and Xu, 2007; Ragozina, 2020; Vankovska, 2020; Biehl *et al.*, 2021; Makarychev and Romashko, 2021), and by extension, biopower (Nguyen, 2009; Ragozina, 2020) and modes of objectification (Lawson and Xu, 2007).

In defining biopower and biopolitics, Foucault writes of the former as ‘an explosion of numerous and diverse techniques for achieving the subjugation of (physical, biological) bodies and the control of populations’ (Liesen and Walsh, 2015), and the latter as the mechanism by which biopower acts—‘the processes by which human life, at the level of the population, emerged as a distinct political problem’, and the extension of states’ power over the bodies of a population, physical and political alike (Means, 2021). In comparison to other articles with a focus on the politics of pandemics, the invocation of biopolitics and biopower is very much focused upon the values of politics rather than the structures it may take, such as legislative coalitions or the leadership of political parties.

We note from the uses of ‘politics’ in the literature we reviewed again some blurring between processes of power relationships and the organizations in which these processes occur or are expressed, such as legislatures and political parties. As an example, again using the distinctions previously set up in discussion on ‘institutions’, a legislature could be seen by itself as not ‘politics’, but instead ‘politics’ as an important determinant of what the legislature does or does not do. In our framing, these processes of where politics and organizations interact is where we locate ‘governance’.

### Governance

Although writing on governance comprised the largest proportion of the reviewed literature (36.9%), in many ways it was also the most varied of the four analysed terms. Contingent upon the terminology used by the authors, writing on governance could similarly be applied to organizations, institutions and politics alike without mention of either term, further underscoring the fluidity with which these four terms have been used in relation to each other in practice.

An example of ‘governance’ being used in relation to both ‘organizations’ and ‘institutions’ can be seen in the notion of structures of governance. Using the definition provided by the *Oxford Handbook of Governance* as ‘the architecture of formal and informal institutions’, structures of governance can be seen as comprised of components such as ‘systems of rules’, ‘regimes of laws, rules, judicial decisions, and administrative practices’, and ‘the comparatively stable institutional, socio-economic and ideational parameters as well as the historically entrenched actor constellations’ (Levi-Faur, 2012). Used in relation to structured organizations such as the WHO (Carney and Bennett, 2014), frameworks such as the IHR (Collins *et al.*, 2021) or the structures of government hierarchies within national jurisdictions (Liu *et al.*, 2020; Wang *et al.*, 2021; Finnane, 2021), this conception of governance is similar to the ways in which ‘organizations’ and ‘institutions’ have been used to characterize the same subject area, and underscores the fluidity with which the four terms of institutions, politics, organizations and governance have been used so far. It may be worth noting that the variety of outcomes experienced in countries with very different organizational and governance structures and processes might argue for some caution in applying these values-based assessments in relation to epidemic and pandemic response.

Additional intersections between conceptions of ‘governance’ and ‘institutions’, and even the use of terms to define each of them, can be seen in descriptions of ‘governance’ as a mechanism and as a process, categorizations both also borrowed from the *Oxford Handbook of Governance.* The former, described as being involved with the ‘institutional procedures of decision-making’^4^, seeks to understand better the ‘naturalization’ of decision making, through mechanisms such as exchanges—monetized or not—, commands in a hierarchy, persuasion and deliberation as well as group identity and loyalty (Levi-Faur, 2012).

The conception of governance as a process builds upon the relatively stable components of structures and adds ‘dynamic interactive aspects’ such as steering and coordinating the ‘practices of governing’ or the ‘exercise of authority’ in policy-making, through which norms of governance are processed as well (Levi-Faur, 2012). Here too there is significant overlap with definitions of governance and institutions, particularly with regard to definitions of the latter which invoke the ‘institutionalization’ of norms within a jurisdiction or acting organization.

Another way in which this prefix tendency arose was the use of the terms themselves as a prefix in a broader phrase, such as ‘institutional rearrangement’, ‘institutional resilience’ (McCormick and Whitney, 2013; Soon *et al.*, 2021; De Angelis and de Oliveira), ‘political systems’ or ‘political participation’ (Burkle, 2020). As seen here, in addition to application with regard to the term ‘governance’, the tendency of applying prefixes to the term has also taken place frequently with ‘politics’, both with regard to values (like ‘good politics’) and descriptions (such as ‘emergency politics’) (Afsahi *et al.*, 2020; Lee, 2020; Ragozina, 2020).

### Implications of this conceptual mapping scoping study for institutions, politics, organizations and governance research

This conceptual mapping scoping study has found a substantial body of research reflecting the importance of IPOG factors as factors affecting responses to public health needs and crises. These terms have been defined by authors in the field, albeit often used without precise definition in individual publications. Before the impact of IPOG factors on epidemic and pandemic responses can be measured, it must be known what these terms mean among researchers in the first place.

This body of literature has also been expanding rapidly in recent years with the COVID-19 pandemic, suggesting increased importance of these factors in this global crisis. However, there is considerable diversity in how these terms are defined, used and related to each other by researchers in the field, which engenders a lack of clarity in describing and analysing phenomena and prescribing action. While some of these trends have been observed in conceptions of governance in health systems more generally (Barbazza and Tello, 2014; NTR *et al.*, 2019), it was found through this research that similar patterns of unclear, varied and competing definitions existed with regard to characterizations of institutions, politics and organizations as well, within the particular context of epidemic and pandemic responses.

As the COVID-19 pandemic marches into its third year, there is little indication that the production of literature on upstream determinants of governments’ response to epidemics and pandemics will substantially subside in the near future. As this volume of literature continues to increase, there will be an increasing need to establish a degree of consistency in the terminology used in order to lessen confusion among researchers, as well as to improve the applicability of new knowledge across a variety of contexts and settings. To this end, a framework such as the IPOG model set forth by the Working Group on Health Systems Response to COVID-19 at the authors’ institute (Berman *et al.*, 2021), mentioned earlier in the Methods section, could be useful in the way it establishes boundaries and strict definitions for each term, as well as in the way in which it clearly sets up concepts and processes to describe how they relate to each other. Wider application of frameworks such as the IPOG model could lead to more consistent analysis of the ways in which IPOG has shaped responses to epidemics and pandemics across jurisdictions.

### Limitations

A key limitation of this research is the limited ability by the reviewers to keep pace with the rate at which literature on government systems’ response to infectious disease outbreaks has been published in the past 3 years. As previously mentioned in the Discussion, literature on COVID-19 has outpaced work published on other diseases (Ioannidis *et al.*, 2021). Similar results have been borne out in this review as well, where included literature on COVID-19 exceeded works on all other diseases combined. We have sought to account for the recent frequency of publication on this topic by conducting an updated search with identical keywords and an updated timeframe that covers the time from which the initial search was conducted in late July 2021 to the time of submission in April 2022. Results from this updated search have since been included in this study. The previously mentioned functional limitations on some of the databases, which limited searches on studies published prior too July 2011 to PAIS and Ovid Medline, are worth mentioning here as well.

Furthermore, even though our search was inclusive of grey literature, a systematized search directed towards grey literature specifically was not undertaken. Thus, it is likely that a wide variety of grey literature relevant to IPOG factors, from sources such as governments, policy briefs and organizational documents, were not indexed in the searched databases, and thus missed without a systematic search for grey literature unto itself. Similar difficulties may have been encountered with book chapters and edited volumes, which can be difficult to index in databases. Even with this limitation, some grey literature sources have been included in this research, and the databases used in the searches were chosen with the intention of capturing a wide variety of literature from a multitude of sources.

To assist with searching, the terms ‘epidemic’ and ‘pandemic’ were both used concurrently with each other. Although beyond the scope of the present study, further research could more thoroughly explore the extent to which IPOG terms are used or conceptualized differently in the event of epidemics or pandemics. Reviewers also noted interest in exploring how this growing literature might be analysed by sub-themes as categories within the IPOG factors as well as how multiple IPOG factors might be referenced together in some literature. We hope this work will stimulate further investigation of this type.

Finally, by restricting our search to the English language, we may also have excluded relevant literature from low- and middle-income countries (e.g. French articles from Western African countries; Spanish or Portuguese articles from Latin American countries).

## Conclusion

### Looking forward

The results from this research have shown that even as interest in the impact of IPOG factors has increased sharply in the past 3 years during the course of the COVID-19 pandemic, inconsistency and disputes continue to characterize the use and definitions of such terms, generating confusion. The results of this study confirm previous findings to this effect on the state of definitions of governance and in health systems more generally (Levi-Faur, 2012; Barbazza and Tello, 2014; NTR *et al.*, 2019); this study extends such findings towards writing on the other three terms, and in the context of epidemic and pandemic response as well.

Already, retrospectives on the COVID-19 pandemic have underscored the need among public health researchers and practitioners to better understand the interactions between public health measures and conceptions of politics (Greer *et al.*, 2021) and governance (Tam, 2021a) in order to ensure the science behind public health measures is effectively used during the next pandemic (Frieden *et al.*, 2021). For example, the PHAC, in its ‘Vision to Transform Canada’s Public Health System’ (Tam, 2021a), has identified ‘effective governance across jurisdictions and sectors’ as not only a principle element of a ‘world-class public health system’ (Tam, 2021b), but also as a research priority going forward (Tam, 2021c). Interest in IPOG terms has also been demonstrated prior to the COVID-19 pandemic through organs such as the United Nations, which includes ‘strong institutions’ as part of its Sustainable Development Goals (Goal 16: Promote just, peaceful and inclusive societies).

The dramatic demands placed on public health organizations during the COVID-19 pandemic has also illustrated the wide variety of organizational structures existing in different jurisdictions. This includes the spectrum of more and less centralized structures in both unitary and federal states. It also includes the role of medical care funders and providers in relation to organizations charged with a greater population health focus and the locus and structure of national institutions of public health such as centres for disease control.

This review was performed to support a wider programme of research, which in our conceptualization focuses more attention on decision-making processes (governance) at the locus between political and organizational factors and influenced by broader contextual factors such as institutions and other contextual factors. We hope that this focus enriches our understanding of how IPOG determinants affect system-responses to public health crises. Learning more about these processes may also contribute to better system designs and the laws and regulations that define them.

As the world reflects on its recent and ongoing experiences with COVID-19, there is growing awareness of the importance of the ‘upstream’ factors discussed in this research. For research to contribute to achieving better outcomes in future crises more investigations of these factors may be needed. Increased attention will also benefit from greater clarity about what is being studied. More explicit definitions of IPOG in terms of their distinct concepts and properties and their interactions in the context of epidemic and pandemic response would be a step in the right direction.

## Supplementary Material

czac091_Supp

## Endnotes

1. As defined in our broader case study protocol (Brubacher *et al.*, 2022), institutions are defined as the formal and informal rules and practices that shape human interactions in a society giving rise to meaning, norm and appropriateness of behaviour; politics as the arena in societies where power is assigned and distributed and where important influences on decision-making regarding health and other topics of public interest occur; organizations refer to governmental public bodies, especially the organizations charged with public-health functions, as well as others in the health system; and governance as the decision-making processes occurring at the interface between politics and organizations, conditioned by institutional norms.

2. As in, uses the exact term(s) (institutions, politics, organizations and/or governance) or employs similar terms or concepts as those defined in our broader case study protocol (Brubacher *et al.*, 2022).

3. This accounts for why the breakdown of articles per term adds to more than 65 included articles.

4. Here, it appears that the term ‘institutions’ is being used to refer to ‘organizations’ in the *Oxford Handbook of Governance*, compared with the use of ‘institutions’ to instead refer to norms, also in the same text.

## Reflexivity statement

The majority of the authors who contributed to this research are women, who provided significant guidance in its development. The authors’ background in public policy and the health sciences provided the authors with both familiarity and awareness of technical terminology and concepts in the reviewed articles. Furthermore, L.J.B.’s background in knowledge synthesis studies informed this research. While many members of the team are part of minority ethnic and religious groups, immigrants to Canada or recent descendants of immigrants to Canada, we also recognize that we are all guests on the land upon which our work at the University of British Columbia’s Vancouver campus was conducted, which is the traditional, ancestral and unceded territory of the Musqueam people.

Even as this research includes perspectives involving all permanently inhabited regions of the world, its writing in English and the study of contemporary conceptions of statehood necessarily privileges western understandings of the role of the state in society, at the expense of alternate perspectives.

The authors are aware of the Eurocentric biases present in published academic literature. Although search criteria inclusive of all geographic regions have been developed, these biases are a limitation of this study and of the literature writ large.

## References

[R1] Adeel AB, Catalano M, Catalano O et al. 2020. COVID-19 policy response and the rise of the sub-national governments. *Canadian Public Policy* 46: 565. 10.3138/cpp.2020-101 PMC9400820 36039151

[R2] Afsahi A, Beausoleil E, Dean R et al. 2020. Democracy in a global emergency five lessons from the COVID-19 pandemic. *Democratic Theory-An Interdisciplinary Journal* 7: V–XIX.

[R3] Agartan TI, Cook S, Lin V. 2020. Introduction: COVID-19 and WHO: global institutions in the context of shifting multilateral and regional dynamics. *Global Social Policy* 20: 367–73.

[R4] Anderson S, Allen P, Peckham S et al. 2008. Asking the right questions: scoping studies in the commissioning of research on the organisation and delivery of health services. *Health Research Policy and Systems* 6: 7. 10.1186/1478-4505-6-7 PMC2500008 18613961

[R5] Areal AG, Sheppy B. 2021. A Crisis of Governance - Or an Opportunity? *Health services insights* 14: 11786329211033844. 10.1177/11786329211033845 PMC8299892 34366672

[R6] Barbazza E, Tello J. 2014. A review of health governance: definitions, dimensions and tools to govern. *Health Policy* 116: 1–11. 24485914 10.1016/j.healthpol.2014.01.007

[R7] Bardosh KL, Scoones JC, Grace D et al. 2017. Engaging research with policy and action: what are the challenges of responding to zoonotic disease in Africa? *Philosophical Transactions: Biological Sciences* 372: 1–10. 10.1098/rstb.2016.0172 PMC5468697 28584180

[R8] Béland D, Cantillon B, Hick R et al. 2021. Social policy in the face of a global pandemic: policy responses to the COVID −19 crisis. *Social Policy and Administration* 55: 249–60. 34230721 10.1111/spol.12718 PMC8251102

[R9] Berman P, Keidar S, Zahid M et al. 2021. Same disease, similar measures, varied outcomes: research to improve understanding of why results in curbing COVID-19 has been so different across jurisdictions around the world? *University of British Columbia Medical Journal* 13: 7–8.

[R10] Biehl J, Prates LEA, Amon JJ. 2021. Supreme Court v. Necropolitics: the chaotic judicialization of COVID-19 in Brazil. *Health and Human Rights* 23: 151. PMC8233022 34194209

[R11] Boyce MR, Katz R. 2021. COVID-19 and the proliferation of urban networks for health security. *Health Policy and Planning* 36: 357–9. 33491068 10.1093/heapol/czaa194 PMC7928945

[R12] Brahmbhatt M, Jonas O. 2015. International cooperative responses to pandemic threats: a critical analysis. *The Brown Journal of World Affairs* 21: 164–79.

[R13] Brandt A, Gardner M. 2000. Antagonism and accommodation: interpreting the relationship between public health and medicine in the United States during the 20th Century. *American Journal of Public Health* 90: 707–15. 10800418 10.2105/ajph.90.5.707 PMC1446218

[R14] Braun V, Clarke V. 2006. Using thematic analysis in psychology. *Qualitative Research in Psychology* 3: 77–101.

[R15] Brubacher LJ, Hasan MZ, Sriram V et al. 2022. Investigating the influence of institutions, politics, organizations, and governance on the COVID-19 response in British Columbia, Canada: a jurisdictional case study protocol. *Health Research Policy and Systems* 20: 74. 10.1186/s12961-022-00868-5 PMC9210337 35729534

[R16] Burkle FM . 2020. Declining public health protections within autocratic regimes: impact on global public health security, infectious disease outbreaks, epidemics, and pandemics. *Prehospital and Disaster Medicine* 35: 237–46. 32238221 10.1017/S1049023X20000424 PMC7156578

[R17] Calain P, Abu Sa’Da C. 2015. Coincident polio and Ebola crises expose similar fault lines in the current global health regime. *Conflict and Health* 9: 29. 10.1186/s13031-015-0058-1 PMC4572646 26380580

[R18] Carney T, Bennett B. 2014. Framing pandemic management: new governance, science or culture? *Health Sociology Review* 23: 136–47.

[R19] Choi H, Kim S-Y, Kim J-W et al. 2021. Mainstreaming of health equity in infectious disease control policy during the COVID-19 pandemic Era. *Journal of Preventive Medicine and Public Health* 54: 1–7. 33618493 10.3961/jpmph.20.593 PMC7939756

[R20] Christensen T, Lægreid P. 2020. Balancing governance capacity and legitimacy: how the norwegian government handled the COVID−19 crisis as a high performer. *Public Administration Review* 80: 774–9. 32836445 10.1111/puar.13241 PMC7280699

[R21] Coco A, Dias T. 2020. Prevent, respond, cooperate states’ due diligence duties vis-a-vis the covid-19 pandemic. *Journal of International Humanitarian Legal Studies* 11: 218–36.

[R22] Collins T, Tello J, Van Hilten M et al. 2021. Addressing the double burden of the COVID-19 and noncommunicable disease pandemics: a new global governance challenge. *International Journal of Health Governance* 26: 199–212.

[R23] Craig P, Ruggerio ED, Frohlich K et al. Taking account of context in population health intervention research: guidance for producers, users and funders of research. Southampton: Canadian Institutes of Health Research-National Institute for Health Research, 2018.

[R24] De Angelis G, de Oliveira E. 2021. COVID-19 and the “state of exception”: assessing institutional resilience in consolidated democracies - a comparative analysis of Italy and Portugal. *Democratization* 28: 1602–21.

[R25] Dostal JM . 2020. Governing under pressure: German policy making during the coronavirus crisis. *The Political Quarterly* 91: 542–52. 32836412 10.1111/1467-923X.12865 PMC7361861

[R26] Doyle JS . 2006. An international public health crisis: can global institutions respond effectively to HIV/AIDS? *Australian Journal of International Affairs* 60: 400–11.

[R27] Everts J . 2013. Announcing Swine Flu and the interpretation of pandemic anxiety. *Antipode* 45: 809–25. 32313327 10.1111/j.1467-8330.2012.01021.x PMC7161815

[R28] Fairchild A, Rosner D, Colgrove J et al. 2010. The EXODUS of public health what history can tell us about the future. *American Journal of Public Health* 100: 54–63. 19965565 10.2105/AJPH.2009.163956 PMC2791244

[R29] Fei D . 2022. Assembling Chinese health engagement in Africa: structures, strategies and emerging patterns. *Third World Quarterly* 43: 1093–114.

[R30] Finnane M . 2021. Governing in a Pandemic: law and Government in Australia, 1919. *Australian Historical Studies* 53: 266–83.

[R31] Forestier C, Cox AT, Horne S. 2016. Coordination and relationships between organisations during the civil-military international response against Ebola in Sierra Leone: an observational discussion. *Journal of the Royal Army Medical Corps* 162: 156–62. 27016507 10.1136/jramc-2015-000612

[R32] Forum on Medical and Public Health Preparedness for Catastrophic Events, Forum on Drug Discovery D and Translation, Forum on Microbial Threats et al. 2016. Rapid medical countermeasure response to infectious diseases.

[R33] Foucault M . 1991. Chapter four: governmentality. In: Burchell G, Gordon C and Miller P (eds). *The Foucault Effect: Studies in Governmentality, with Two Lectures by and an Interview with Michel Foucault*. Chicago: The University of Chicago Press, 87–104.

[R34] Fowler L, Kettler J, Witt S. 2021. Pandemics and partisanship: following old paths into uncharted territory. *American Politics Research* 49: 3–16.

[R35] Frieden T, Buissonnière M, McClelland A. 2021. The world must prepare now for the next pandemic. *BMJ Global Health* 6: e005184. 10.1136/bmjgh-2021-005184 PMC7970315 33727280

[R36] GBD 2019 Diseases and Injuries Collaborators . 2020. Global burden of 369 diseases and injuries in 204 countries and territories, 1990–2019: a systematic analysis for the Global Burden of Disease Study 2019. *The Lancet* 396: 1204–22. 10.1016/S0140-6736(20)30925-9 PMC7567026 33069326

[R37] Gerwin LE . 2011. Planning for pandemic: a new model for governing public health emergencies. *American Journal of Law & Medicine* 37: 128–71. 21614997 10.1177/009885881103700104

[R49] Goal 16: promote just, peaceful and inclusive societies. *United Nations*. https://www.un.org/sustainabledevelopment/peace-justice/.

[R38] Goetz KH, Martinsen DS, 2021. COVID-19: a dual challenge to European liberal democracy. *West European Politics* 44:1003–24.

[R39] Gostin LO, Katz R. 2016. The international health regulations: the governing framework for global health security. *The Milbank Quarterly* 94: 264–313. 27166578 10.1111/1468-0009.12186 PMC4911720

[R40] Greer SL, King EJ, Massard da Fonseca E et al. 2021. *Coronavirus Politics: The Comparative Politics and Policy of COVID-19*. Ann Arbor: University of Michigan Press.

[R41] Greer SL, Singer PM. 2017. Addressing Zika in the United States: polarization, fragmentation, and public health. *American Journal of Public Health* 107: 861–2. 28498743 10.2105/AJPH.2017.303772 PMC5425874

[R42] Hale T, Anania J, Mello BA de et al. 2022. *Variation in Government Responses to COVID-19 Version 13.0*. Oxford: University of Oxford Blavatnik School of Government.

[R43] Hanrieder T, Kreuder-Sonnen C. 2014. WHO decides on the exception? Securitization and emergency governance in global health. *Security Dialogue* 45: 331–48.

[R44] He A, Shi Y, Liu H. 2020. Crisis governance, Chinese style: distinctive features of China’s response to the Covid-19 pandemic. *Policy Design and Practice* 3: 242–58.

[R45] Hier S . 2021. Narrating the crisis: moral regulation, overlapping responsibilities and COVID-19 in Canada. *Current Sociology*.

[R46] Hirsch JS, Giang LM, Parker RG et al. 2015. Caught in the middle: the contested politics of HIV/AIDS and health policy in Vietnam. *Journal of Health Politics, Policy and Law* 40: 13. 10.1215/03616878-2854447 PMC4352397 25480849

[R47] Hoffman SJ . 2010. The evolution, etiology and eventualities of the global health security regime. *Health Policy and Planning* 25: 510–22. 20732860 10.1093/heapol/czq037

[R48] Honigsbaum M . 2017. Between securitisation and neglect: managing ebola at the borders of global health. *Medical History* 61: 270–94. 28260567 10.1017/mdh.2017.6 PMC5426310

[R50] Huang K . 2021. Minimizing the social dilemma. *Investigación Económica* 80: 32–55.

[R51] Huang H, Peng Z, Wu H et al. 2020. A big data analysis on the five dimensions of emergency management information in the early stage of COVID-19 in China. *Journal of Chinese Governance* 5: 213–33.

[R52] Ioannidis J, Salholz-Hillel M, Boyack K et al. 2021. The rapid, massive growth of COVID-19 authors in the scientific literature. *Royal Society Open Science* 8: 210389. 10.1098/rsos.210389 PMC8422596 34527271

[R53] Jones E, MacDougall H, Monnais L et al. 2021. Beyond the COVID-19 crisis: building on lost opportunities in the history of public health. Ottawa: Royal Society of Canada *Facets* 6.

[R54] Jönsson C, Jönsson K. 2012. Global and local health governance: civil society, human rights and HIV/AIDS. *Third World Quarterly* 33: 1719–34.

[R55] Kamradt-Scott A . 2013. The politics of medicine and the global governance of pandemic influenza. *International Journal of Health Services* 43: 105–21. 23527457 10.2190/HS.43.1.h

[R56] Kim YS . 2015. World health organization and early global response to HIV/AIDS: emergence and development of international norms. *Journal of International and Area Studies* 22: 19–40.

[R57] Kim M-H, Cho W, Choi H et al. 2020a. Assessing the South Korean model of emergency management during the COVID-19 pandemic. *Asian Studies Review* 44: 567–78.

[R58] Kim Y, Oh S, Wang C. 2020b. From uncoordinated patchworks to a coordinated system: MERS-CoV to COVID-19 in Korea. *The American Review of Public Administration* 50: 736–42.

[R59] Lawson J, Xu F. 2007. SARS in Canada and China: two approaches to emergency health policy. *Governance: An International Journal of Policy, Administration, and Institutions* 20: 209–32.

[R60] Lee K . 2020. WHO under fire: the need to elevate the quality of politics in global health. *Global Social Policy* 20: 374–7.

[R61] Levi-Faur D . 2012. From “big government” to “big governance”? In: Levi-Faur D (ed). *The Oxford Handbook of Governance*. Oxford: Oxford University Press, 3–18.

[R62] Liesen L, Walsh B. 2015. The competing meanings of “biopolitics” in political science: biological and postmodern approaches to politics. *Politics and the Life Sciences* 31: 2–15. 10.2990/31_1-2_2 23379312

[R63] Liu Y . 2004. China’s public health-care system: facing the challenges. *World Health Organization Bulletin of the World Health Organization* 82: 532–8. 15500285 PMC2622899

[R64] Liu Z, Guo J, Zhong W et al. 2021. Multi-level governance, policy coordination and subnational responses to COVID-19: comparing China and the US. *Journal of Comparative Policy Analysis: Research and Practice* 23: 204–18.

[R65] Liu P, Zhong X, Yu S. 2020. Striking a balance between science and politics: understanding the risk-based policy-making process during the outbreak of COVID-19 epidemic in China. *Journal of Chinese Governance* 5: 198–212.

[R66] Mack A, Snair MR, Choffnes E. 2016. Global Health Risk Framework: Governance for Global Health Workshop Summary, https://nap.nationalacademies.org/catalog/21854/global-health-risk-framework-governance-for-global-health-workshop-summary. 26962612

[R67] Makarychev A, Romashko T. 2021. Precarious sovereignty in a post-liberal Europe: the COVID-19 emergency in Estonia and Finland. *Chinese Political Science Review* 6: 63–85. 10.1007/s41111-020-00165-y PMC7581947 40477106

[R68] McCormick S, Whitney K. 2013. The making of public health emergencies: West Nile virus in New York City. *Sociology of Health & Illness* 35: 268–79. 23278188 10.1111/1467-9566.12002

[R69] Means A . 2021. Foucault, biopolitics, and the critique of state reason. *Educational Philosophy and Theory* 54: 1968–9.

[R70] Meszaros G . 2021. Carl schmitt in hungary: constitutional crisis in the shadow of Covid-19. *Review of Central and East European Law* 46: 69–90.

[R71] Moulds S . 2020. Scrutinising COVID-19 laws: an early glimpse into the scrutiny work of federal parliamentary committees. *Alternative Law Journal* 45: 180.

[R72] Nelson MJ . 2021. Pandemic politics in South Asia: muslims and democracy. *The Review of Faith & International Affairs* 19: 83–94.

[R73] Nguyen VK . 2009. Government-by-exception: enrolment and experimentality in mass HIV treatment programmes in Africa. *Social Theory & Health* 7: 196–217.

[R74] Nohrstedt D, Baekkeskov E. 2018. Political drivers of epidemic response: foreign healthcare workers and the 2014 Ebola outbreak. *Disasters* 42: 41–61. 28440550 10.1111/disa.12238

[R75] NTR A, AM R, PC L. 2019. The literature review of the governance frameworks in health system. *Journal of Public Administration and Governance* 9: 252–60.

[R76] Peters MDJ, Godfrey CM, Khalil H et al. 2015. Guidance for conducting systematic scoping reviews. *International Journal of Evidence-Based Healthcare* 13: 141–6. 26134548 10.1097/XEB.0000000000000050

[R77] Potter S . 1964. *Jacobellis v. Ohio*. 378 U.S. at 197 (Stewart, J., concurring), specifically about passage “I know it when I see it.” https://www.law.cornell.edu/supremecourt/text/378/184.

[R78] Ragozina S . 2020. Islamic Biopolitics during Pandemics in Russia: Intertextuality of Religious, Medical and Political Discourses. *Anthropology in Action-Journal for Applied Anthropology in Policy and Practice* 27: 82–6.

[R79] Rocco P, Beland D, Waddan A. 2020. Stuck in neutral? Federalism, policy instruments, and counter-cyclical responses to COVID-19 in the United States. *Policy and Society* 39: 458–77. 35039731 10.1080/14494035.2020.1783793 PMC8754696

[R80] Roodenrijs JCM, Kraaij-Dirkzwager MM, van den Kerkhof JHTC et al. 2014. Risk governance for infectious diseases: exploring the feasibility and added value of the IRGC-framework for Dutch infectious disease control. *Journal of Risk Research* 17: 1161–82.

[R81] Rosella L, Wilson K, Crowcroft N et al. 2013. Pandemic H1N1 in Canada and the use of evidence in developing public health policies - A policy analysis. *Social Science & Medicine* 83: 1–9. 23465198 10.1016/j.socscimed.2013.02.009 PMC7125641

[R82] Sandset T . 2021. The necropolitics of COVID-19: race, class and slow death in an ongoing pandemic. *Global Public Health* 16: 1411–23. 33761846 10.1080/17441692.2021.1906927

[R83] Sanyaolu A, Okorie C, Hosein Z et al. 2021. Global pandemicity of COVID-19: situation report as of June 9, 2020. *Infectious Diseases: Research and Treatment* 14. 10.1177/1178633721991260 PMC7863149 33597811

[R84] Schwartz J, Evans RG, Greenberg S. 2007. Evolution of Health Provision in Pre-SARS China: The Changing Nature of Disease Prevention. *China Review* 7: 81–104.

[R85] Shultz L, Viczko M. 2021. What are we saving? Tracing governing knowledge and truth discourse in global COVID-19 policy responses. *International Review of Education* 67: 219–39. 34024934 10.1007/s11159-021-09893-y PMC8130215

[R86] Soon S, Chou CC, Shi S. 2021. Withstanding the plague: institutional resilience of the East Asian welfare state. *Social Policy and Administration* 55: 374–87. 33821059 10.1111/spol.12713 PMC8014878

[R87] Speakman EM, Burris S, Coker R. 2017. Pandemic legislation in the European Union: fit for purpose? The need for a systematic comparison of national laws. *Health Policy* 121: 1021. 10.1016/j.healthpol.2017.08.009 28935231

[R88] Statistics Division of the United Nations Secretariat. *Methodology: Standard country or area codes for statistical use (M49)*. Methodology. https://unstats.un.org/unsd/methodology/m49/.

[R89] Tam T . 2021a. The chief public health officer of Canada’s Report on the State of Public Health in Canada 2021: a vision to transform canada’s public health system. Ottawa: Public Health Agency of Canada, 129.

[R90] Tam T Report summary: a vision to transform Canada’s public health system. Ottawa: Public Health Agency of Canada, 2021b:12.

[R91] Tam T . 2021c. Generating knowledge to inform public health system transformation. Ottawa: Public Health Agency of Canada, 7.

[R92] Teigen PM . 2007. Legislating fear and the public health in gilded age Massachusetts. *Journal of the History of Medicine and Allied Sciences* 62: 141–70. 16980330 10.1093/jhmas/jrl016

[R93] Thomson S, Ip EC. 2020. COVID-19 emergency measures and the impending authoritarian pandemic. *Journal of Law and the Biosciences* 7: lsaa064. 10.1093/jlb/lsaa064 PMC7543595 33569176

[R94] Tricco AC, Lillie E, Zarin W et al. 2018. PRISMA extension for scoping reviews (PRISMA-ScR): checklist and explanation. *Annals of Internal Medicine* 169: 467–73. 30178033 10.7326/M18-0850

[R95] US Department of Health and Human Services . 2021. What is the U.S. Opioid Epidemic?

[R96] Vankovska B . 2020. Dealing with COVID-19 in the European periphery: between securitization and “gaslighting”. *Journal of Global Faultlines* 7: 71–88.

[R97] Wang C, Dong X, Zhang Y et al. 2021. Community resilience governance on public health crisis in China. *International Journal of Environmental Research and Public Health* 18: 2123. 10.3390/ijerph18042123 PMC7926474 33671618

[R98] Wenham C . 2018. Regionalizing health security. *Contemporary Southeast Asia* 40: 126–51.

[R99] West-Oram P . 2021. Solidarity is for other people: identifying derelictions of solidarity in responses to COVID-19. *Journal of Medical Ethics* 47: 65–8. 32647044 10.1136/medethics-2020-106522

[R100] Wilson K, Lazar H. 2006. From SARS to Avian flu—why Ottawa must lead Canada’s response. *Policy Options/Options Politiques* 27: 31–8.

[R101] Windholz E . 2020. Governing in a pandemic: from parliamentary sovereignty to autocratic technocracy. *The Theory and Practice of Legislation* 8: 93–113.

[R102] Wu A . 2022. *Review of the Book Coronavirus Politics: The Comparative Politics and Policy of COVID-19*, by Greer S et al. Hoboken, New Jersey: WMHP.

[R103] Yan B, Chen B, Wu L et al. 2021. Culture, institution, and COVID-19 first-response policy: a qualitative comparative analysis of thirty-one countries. *Journal of Comparative Policy Analysis: Research and Practice* 23: 219–33.

[R104] Yao D, Li J, Chen Y et al. 2021. Interactive governance between and within governmental levels and functions: a social network analysis of China’s case against COVID-19. *American Review of Public Administration* 52: 191–205. 10.1177/02750740211059534 PMC8883134 35382106

[R105] Zhang X, Wang L. 2021. Administrative governance and frontline officers in the chinese prison system during the COVID-19 pandemic. *Asian Journal of Criminology* 16: 1–17. 33727986 10.1007/s11417-021-09345-4 PMC7952142

[R106] Zhang J and Zhang R. 2020. COVID-19 in China: Power, Transparency and Governance in Public Health Crisis. *Healthcare* 8: 288. 10.3390/healthcare8030288 PMC7551406 32842607

[R107] Zhou S, Han L, Liu P et al. 2020. Global health governance for travel health: lessons learned from the coronavirus disease 2019 (COVID-19) outbreaks in large cruise ships. *Global Health Journal* 4: 133–8. 33294249 10.1016/j.glohj.2020.11.006 PMC7709727

