# Supplementary material for: How have researchers defined institutions, politics, organizations and governance in research related to epidemic and pandemic response? A scoping review to map current concepts
Source: Health Policy Plan. 2022 Oct 29;38(3):377–93. doi: 10.1093/heapol/czac091 (PMC9620353; doi:10.1093/heapol/czac091)
Supplement: czac091_Supp [file czac091_supp.zip › Supplementary Material_revised_clean_arabic.docx]

**APPENDIX 1.** Reporting Items for Systematic reviews and Meta-Analyses extension for Scoping Reviews (PRISMA-ScR) Checklist

| **SECTION** | **ITEM** | **PRISMA-ScR CHECKLIST ITEM** | **REPORTED ON PAGE #** |
| --- | --- | --- | --- |
| **TITLE** | | | |
| Title | 1 | Identify the report as a scoping review. | #1 |
| **ABSTRACT** | | | |
| Structured summary | 2 | Provide a structured summary that includes (as applicable): background, objectives, eligibility criteria, sources of evidence, charting methods, results, and conclusions that relate to the review questions and objectives. | #1 |
| **INTRODUCTION** | | | |
| Rationale | 3 | Describe the rationale for the review in the context of what is already known. Explain why the review questions/objectives lend themselves to a scoping review approach. | #2 |
| Objectives | 4 | Provide an explicit statement of the questions and objectives being addressed with reference to their key elements (e.g., population or participants, concepts, and context) or other relevant key elements used to conceptualize the review questions and/or objectives. | #2 |
| **METHODS** | | | |
| Protocol and registration | 5 | Indicate whether a review protocol exists; state if and where it can be accessed (e.g., a Web address); and if available, provide registration information, including the registration number. | #4 #18 |
| Eligibility criteria | 6 | Specify characteristics of the sources of evidence used as eligibility criteria (e.g., years considered, language, and publication status), and provide a rationale. | #3 #4 |
| Information sources* | 7 | Describe all information sources in the search (e.g., databases with dates of coverage and contact with authors to identify additional sources), as well as the date the most recent search was executed. | #3 |
| Search | 8 | Present the full electronic search strategy for at least 1 database, including any limits used, such that it could be repeated. | #3 |
| Selection of sources of evidence† | 9 | State the process for selecting sources of evidence (i.e., screening and eligibility) included in the scoping review. | #4 |
| Data charting process‡ | 10 | Describe the methods of charting data from the included sources of evidence (e.g., calibrated forms or forms that have been tested by the team before their use, and whether data charting was done independently or in duplicate) and any processes for obtaining and confirming data from investigators. | #4 |
| Data items | 11 | List and define all variables for which data were sought and any assumptions and simplifications made. | Appendix (Supplementary Material) |
| Critical appraisal of individual sources of evidence§ | 12 | If done, provide a rationale for conducting a critical appraisal of included sources of evidence; describe the methods used and how this information was used in any data synthesis (if appropriate). | #3 |
| Synthesis of results | 13 | Describe the methods of handling and summarizing the data that were charted. | #3 and #4 |
| **RESULTS** | | | |
| Selection of sources of evidence | 14 | Give numbers of sources of evidence screened, assessed for eligibility, and included in the review, with reasons for exclusions at each stage, ideally using a flow diagram. | #5 |
| Characteristics of sources of evidence | 15 | For each source of evidence, present characteristics for which data were charted and provide the citations. | #5 |
| Critical appraisal within sources of evidence | 16 | If done, present data on critical appraisal of included sources of evidence (see item 12). | #10 and #11 |
| Results of individual sources of evidence | 17 | For each included source of evidence, present the relevant data that were charted that relate to the review questions and objectives. | #5 |
| Synthesis of results | 18 | Summarize and/or present the charting results as they relate to the review questions and objectives. | #5 #6 #7 #8 #9 |
| **DISCUSSION** | | | |
| Summary of evidence | 19 | Summarize the main results (including an overview of concepts, themes, and types of evidence available), link to the review questions and objectives, and consider the relevance to key groups. | #10 |
| Limitations | 20 | Discuss the limitations of the scoping review process. | #15 |
| Conclusions | 21 | Provide a general interpretation of the results with respect to the review questions and objectives, as well as potential implications and/or next steps. | #15 |
| **FUNDING** | | | |
| Funding | 22 | Describe sources of funding for the included sources of evidence, as well as sources of funding for the scoping review. Describe the role of the funders of the scoping review. | Title Page #2 |

JBI = Joanna Briggs Institute; PRISMA-ScR = Preferred Reporting Items for Systematic reviews and Meta-Analyses extension for Scoping Reviews.

* Where *sources of evidence* (see second footnote) are compiled from, such as bibliographic databases, social media platforms, and Web sites.

† A more inclusive/heterogeneous term used to account for the different types of evidence or data sources (e.g., quantitative and/or qualitative research, expert opinion, and policy documents) that may be eligible in a scoping review as opposed to only studies. This is not to be confused with *information sources* (see first footnote).

‡ The frameworks by Arksey and O’Malley (6) and Levac and colleagues (7) and the JBI guidance (4, 5) refer to the process of data extraction in a scoping review as data charting*.*

§ The process of systematically examining research evidence to asses#s its validity, results, and relevance before using it to inform a decision. This term is used for items 12 and 19 instead of "risk of bias" (which is more applicable to systematic reviews of interventions) to include and acknowledge the various sources of evidence that may be used in a scoping review (e.g., quantitative and/or qualitative research, expert opinion, and policy document).

*From:* Tricco AC, Lillie E, Zarin W, O'Brien KK, Colquhoun H, Levac D, et al. PRISMA Extension for Scoping Reviews (PRISMAScR): Checklist and Explanation. Ann Intern Med. 2018;169:467–473. [doi: 10.7326/M18-0850](http://annals.org/aim/fullarticle/2700389/prisma-extension-scoping-reviews-prisma-scr-checklist-explanation)

**Appendix 2.** Full search strings used in each database, and number of records retrieved.

JSTOR

| Category | Terms |
| --- | --- |
| IPOG terms | (Institution* OR norm* OR legal OR regulat* OR enforc* OR organi?ation OR politc* OR decision OR ideolog* OR elect* OR power OR policy OR decision OR sociopolitical OR govern*) [Abstract] AND |
| Health Crisis terms | Pandemic [Abstract] |
| Exclusions | N/A |
| Miscellaneous | ‘Content I can access’, Articles and Research Reports, English |
| Time frame | 26 July 2011 to 26 July 2021  26 April 2021 to 8 April 2022 |

PAIS

| Category | Terms |
| --- | --- |
| IPOG terms | (Institution* OR norm* OR conduct OR legal OR regulat* OR enforc* OR organi?ation OR politic* OR decision OR ideolog* OR official* OR elect* OR power OR policy OR decision OR sociopolitical OR govern*) [Anywhere except full text] AND |
| Health Crisis terms | (Pandemic OR ‘infectious disease event’ OR epidemic) [Anywhere except full text]  AND  (Emergency OR ‘state of’ OR ‘declaration of’ OR ‘public health crisis’ OR ‘public health emergency’) [Anywhere except full text] |
| Exclusions | NOT (opioid OR opinion OR pre-pandemic OR survey) [Anywhere except full text] |
| Miscellaneous | Source type: Magazines, Reports, and Scholarly Journals  Document type: all  English |
| Time frame | Before 26 July 2011  26 July 2011 to 26 July 2021  26 April 2021 to 8 April 2022 |

Web of Science

| Category | Terms |
| --- | --- |
| IPOG terms | (Institution* OR legal OR enforc* OR organi?ation OR politc* OR ideolog* OR elect* OR policy OR decision OR sociopolitical OR govern* OR regulation* OR regulatory) [Topic] AND |
| Health Crisis terms | (Pandemic* OR ‘infectious disease event’ OR epidemic*) [Topic]  AND  (‘state of emergenc*’ OR ‘declaration adj4 emergenc*’ OR ‘public health crisis’ OR ‘public health emergenc*’ OR ‘state adj4 emergenc*’) [Topic] |
| Exclusions | NOT (opioid OR opinion OR pre-pandemic OR survey) [Topic] |
| Miscellaneous | English |
| Time frame | 26 July 2011 to 26 July 2021  26 April 2021 to 8 April 2022 |

Medline

| Category | Terms |
| --- | --- |
| IPOG terms | (Institution* OR legal OR enforc* OR organi?ation OR politc* OR ideolog* OR elect* OR policy OR decision OR sociopolitical OR govern* OR regulation* OR regulatory) AND |
| Health Crisis terms | (Pandemic* OR ‘infectious disease event’ OR epidemic*)  AND  (‘state of emergenc*’ OR ‘declaration adj4 emergenc*’ OR ‘public health crisis’ OR ‘public health emergenc*’ OR ‘state adj4 emergenc*’) |
| Exclusions | NOT (opioid OR opinion OR pre-pandemic OR survey) |
| Miscellaneous | English |
| Time frame | 1860 to 2011  2011 to 2021  2021 to 2022 |

**Appendix 3.** More specific inclusion and exclusion criteria for Level 1 screening

| **Included** | **Excluded** |
| --- | --- |
| Articles focused on health/risk communication, e.g. between decision-makers, those in positions of authority, and the general public | Articles on ‘the general public’s perceptions’ of pandemic response or of government action  /decisions related to a public health emergency |
| Articles on HIV as related to IPOG factors (considered as a pandemic/public health emergency in this review) | Articles that focus on reporting an emerging public health threat/crisis, which may mention the need for government/organizational action, but not as the focus of the inquiry |
| Articles that are conceptual/theoretical (e.g. not linked to a specific event/emergency response) *if* they are helpful in defining/operationalizing IPOG factors | Articles that are conceptual/theoretical (as a point of inquiry/focus), e.g. using a ‘health and human rights framework’ to conceptualize public health response to emergency; bioethics articles without connection to particular events/people; ‘calls to action’/commentary without analysis of an event/situation |
| Articles on pandemics/epidemics/public health emergencies/spread of infectious illness generally (as related to IPOG & upstream decision-making), including, e.g: pandemic influenza (e.g. H1N1), measles, Severe Acute Respiratory Syndrome (SARS), tuberculosis, Ebola, Zika | Articles on ‘epidemics’ of other kinds, e.g. non-communicable diseases or substance use (e.g. an ‘epidemic’ of childhood obesity or diabetes) |
| Articles that describe the public health and/or government response to an infectious illness/pandemic/epidemic, including significant reference to IPOG factors (e.g. various organizational structures/roles in the response) | Articles on bioterrorism/biological weapons as a public health threat (not focused on *responses* but hypothetical threats not yet occurred) |
| Articles on education or training (e.g. of decision-makers in statutory laws/legal mechanisms relevant to pandemic preparedness) in connection to an actual non-communicable disease event/emergency | Articles on vaccine rationing at the ‘downstream’ level (e.g. among primary healthcare providers, local public health authorities, hospital administrators); vaccine hesitancy/perception among the public; vaccine development (unless focused on policy); or vaccine-injury compensation |
|  | Articles focused on research or the place of research in public health governance and emergency response/preparedness |
|  | Articles on IPOG as related to mental/behavioural health associated with public health emergencies |
|  | Articles on disaster response (not specific to infectious illness/pandemics/epidemics) |
|  | Articles on policy/politics surrounding global health aid for pandemics/public health crises |

**Appendix 4.** Data extraction questions

- Authors
- Year of publication
- Type of source (multiple-selection checkbox)
  - Primary peer-reviewed article
  - Secondary Review
  - Working paper
  - News item/grey literature
  - Editorial/commentary
  - Quantitative
  - Qualitative
  - Mixed methods
  - Other
- Research question
- Study location (multiple-selection checkbox, based upon UN Geoscheme)
  - Africa
  - Americas
  - Asia
  - Europe
  - Oceania
  - Multilateral (Such as the UN, WHO, World Bank, etc.
- Event(s) studied (multiple-selection checkbox)
  - COVID-19
  - Cholera
  - Ebola
  - HIV/AIDS
  - Influenza
  - SARS/MERS
  - Smallpox
  - Tuberculosis
  - Zika
  - Other (text box entry)
- Disciplinary lens (multiple-selection checkbox)
  - Health care
  - History
  - International relations
  - Law
  - Philosophy/theory
  - Political science
  - Public health
  - Other (text box entry)
- IPOG terms defined
  - Institutions
  - Politics
  - Organizations
  - Governance
  - Other
- Definitions used of IPOG terms (text box entry)
- Impact of IPOG factors on pandemic response (text box entry)
